# Integrated Multi-Omics Analysis Reveals Differential Effects of Fructo-Oligosaccharides (FOS) Supplementation on the Human Gut Ecosystem

**DOI:** 10.3390/ijms231911728

**Published:** 2022-10-03

**Authors:** Tamotsu Kato, Masaharu Kagawa, Wataru Suda, Yuuri Tsuboi, Sayo Inoue-Suzuki, Jun Kikuchi, Masahira Hattori, Toshiko Ohta, Hiroshi Ohno

**Affiliations:** 1 Laboratory for Intestinal Ecosystem, RIKEN Center for Integrative Medical Sciences (IMS), Kanagawa 230-0045, Japan; 2 Immunobiology Laboratory, Graduate School of Medical Life Science, Yokohama City University, Kanagawa 230-0045, Japan; 3 Institute of Nutrition Sciences, Kagawa Nutrition University, Saitama 350-0288, Japan; 4 Laboratory for Microbiome Sciences, RIKEN Center for Integrative Medical Sciences (IMS), Kanagawa 230-0045, Japan; 5 Environmental Metabolic Analysis Research Team, RIKEN Center for Sustainable Resource Sciences (CSRS), Kanagawa 230-0045, Japan; 6 Environmental Factor Analysis Laboratory, Graduate School of Medical Life Science, Yokohama City University, Kanagawa 230-0045, Japan; 7 Graduate School of Advanced Science and Engineering, Waseda University, Tokyo 169-8555, Japan; 8 Japan Aerospace Exploration Agency (JAXA), Ibaraki 305-8505, Japan

**Keywords:** fructo-oligosaccharides, microbiome, gut ecosystem, omics analysis, metabolome

## Abstract

Changes in the gut ecosystem, including the microbiome and the metabolome, and the host immune system after fructo-oligosaccharide (FOS) supplementation were evaluated. The supplementation of FOS showed large inter-individual variability in the absolute numbers of fecal bacteria and an increase in *Bifidobacterium*. The fecal metabolome analysis revealed individual variability in fructose utilization in response to FOS supplementation. In addition, immunoglobulin A(IgA) tended to increase upon FOS intake, and peripheral blood monocytes significantly decreased upon FOS intake and kept decreasing in the post-FOS phase. Further analysis using a metagenomic approach showed that the differences could be at least in part due to the differences in gene expressions of enzymes that are involved in the fructose metabolism pathway. While the study showed individual differences in the expected health benefits of FOS supplementation, the accumulation of “personalized” knowledge of the gut ecosystem with its genetic expression may enable effective instructions on prebiotic consumption to optimize health benefits for individuals in the future.

## 1. Introduction

The commensal microbiota is a complex microbial community that consists of huge numbers of microbes that reside in both mucosal and external surfaces of the human body and their metabolites [1]. Commensal microbiota on the skin, mouth, digestive tract, and vagina are known to have different compositions [2]. With a comparable number to the eukaryotic cells constituting a host human body, gut commensal microbes construct a symbiotic ecosystem with the host [3]. Along with other various functions, the microbiome has been reported to affect the immune function of the host and has also been considered to play an important role in the maintenance of our health [2,4,5].

In the gastrointestinal tract, a vast majority of approximately 1000 bacterial species reside in the colon [6,7,8]. Gut microbiota has been reported to be influenced by a number of factors, including the diet of the host [9,10]. Among dietary components, substrates selectively utilized by host microorganisms to confer a health benefit are defined as prebiotics [11]. Non-digestible oligosaccharides such as fructo-oligosaccharides (FOS) are common prebiotics that particularly assist the growth of lactic acid-producing bacteria species (e.g., *Bifidobacterium* and *Lactobacillus*) [12,13]. FOS are also known to have a number of beneficial effects on the immune system, including increased production of short-chain fatty acids (SCFAs), enhancement of immune responses to lipopolysaccharides (LPS) [14], and also augmented secretion of Immunoglobulin A (IgA) from Peyer’s patch cells [15,16]. However, a vast majority of these findings on FOS supplementation were from animal studies, and results from human subjects are limited. Considering a large inter-individual variability in the human gut microbial composition [2,17] and the presence of bacterial strains that differ in carbohydrate metabolism [18,19,20], it is hypothesized that the effects of FOS supplementation on the human gut ecosystem and its health benefits may vary between individuals. Additionally, there have been scarce studies that use comprehensive approaches to explore the effects of FOS supplementation on the human gut ecosystem, including metabolites and health benefits to the host. Therefore, the present study aimed to evaluate the effects of FOS supplementation on the gut microbiome and immune cells of the host using an integrated multi-omics analysis.

## 2. Results

A total of 11 Japanese males underwent a nine-week longitudinal intervention study with a four-week FOS supplementation. The study period was divided into three distinct phases (i.e., pre, FOS, and post phases). At each phase, samples of feces and blood were collected twice from all participants (Supplementary Figure S1).

### 2.1. Increase of Bifidobacterium in Feces by FOS Supplementation

**Figure 1:**
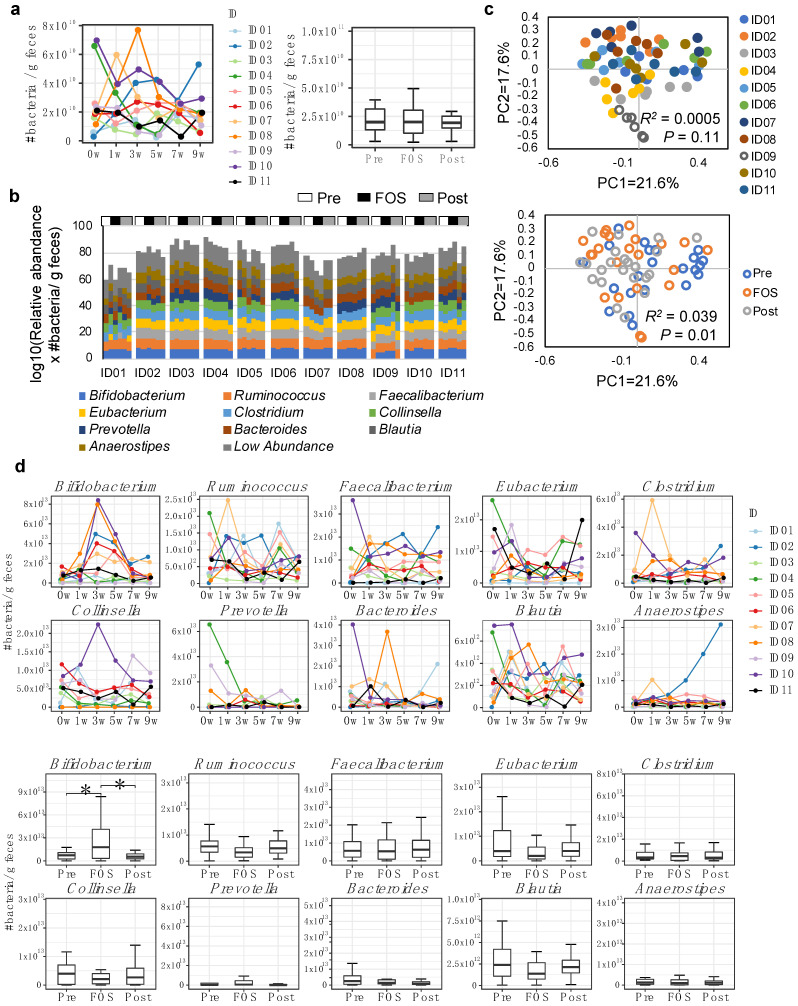
Changes in the total number of bacteria in the fecal microbiota upon FOS supplementation. (**a**) The absolute number of bacteria in each individual by real-time 16S rRNA gene qPCR. (**b**) The composition of microbiota at the genus level in fecal samples using 16S rRNA gene amplicon sequencing. These figures were constructed by the top 10 bacteria at the genus level, respectively. (**c**) PCoA analysis using Bray–Curtis distance of fecal microbiome colored by individuals (**Upper**) and sampling periods (**Lower**). (**d**) The total number of each bacterium in feces at the genus level. The upper figure is illustrated for each individual and the lower figure is summarized for the sample period. * = padj < 0.05.

From 16S rRNA gene amplicon sequencing of extracted bacterial DNAs, fecal samples showed large inter-individual variability in the total number of bacteria and their response to the FOS supplementation that resulted in no significant differences in the total number of bacteria in the feces (Figure 1a). While major bacterial genera that constituted the composition of the microbiome were consistent among participants, the number of bacteria in each genus and their response to the FOS supplementation varied individually (Figure 1b). PCoA using the Bray–Curtis distance based on the 16S rRNA gene amplicon sequencing confirmed the significant inter-individual variabilities of microbiome in the fecal samples (Figure 1c). Similar results were evident when the bacterial composition of fecal microbiota was analyzed based on the relative abundance (Supplementary Figure S3a,b). Among the major bacteria that constitute intestinal microbiota, the number of *Bifidobacterium* was found to be significantly (*p* < 0.05) increased during the FOS supplementation period (Figure 1d). By contrast, when the samples were analyzed based on a relative abundance using the Wilcoxon test with the Benjamini-Hochberg adjustment, although the difference was smaller, a significant increase in *Bifidobacterium* and a significant decrease in *Blautia* were observed during the FOS phase (*p* < 0.05 or *p* < 0.01, Supplementary Figure S3c). A significantly (*p* < 0.05) lower Shannon index at the FOS phase compared with both pre and post phases may indicate an occurrence of decreased fecal microbial diversity as a result of FOS intake (Supplementary Figure S3d). 

### 2.2. Increase of Fecal Fructose during the FOS Intake in Some Individuals

**Figure 2:**
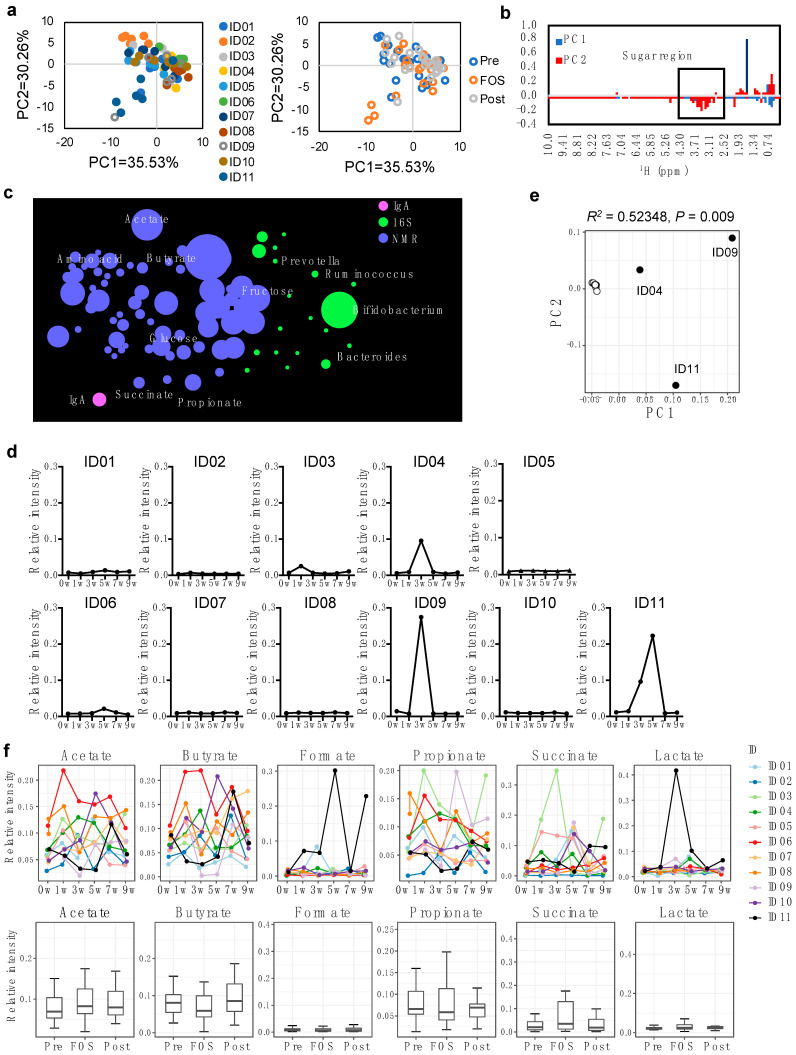
Differences of fecal metabolites upon intake of FOS. (**a**) Score plots of PCA for metabolic profiles of feces colored by individuals (**Left**) and sampling periods (**Right**). (**b**) Loading plot of PCA for fecal metabolome. Blue bars indicate PC1 direction and red bars indicate PC2 direction in the PCA plot in a. PC2 negative direction was influenced by the sugar region (rectangle). (**c**) The correlation network of feces was constructed as described in the Materials and Methods. The line between each element was connected to the calculated correlation coefficient and elements with *p* < 0.05. The size of each element reflects the relative value within each measurement. The amount of fecal IgA was shown in magenta, bacteria in green, and metabolites in blue. (**d**) Relative intensity of fructose in individual fecal samples. (**e**) PCoA based on Euclidean distance of the changes in fructose concentration over time in feces from individuals shown in (**b**). ADONIS revealed that ID04, ID09 and ID11 were significantly different from the others (R2 = 0.52348, *p* = 0.009). (**f**) Relative intensity of fecal SCFAs measured by NMR.

Next, we evaluated the effects of FOS intake on the gut metabolome using nuclear magnetic resonance (NMR)-based measurement of fecal metabolites. We detected 32 metabolites as listed in Table S3. Consistent with the results observed from PCoA on the gut microbiota, principal component analysis (PCA) of NMR-based fecal metabolites analysis showed a large inter-individual variability of gut metabolites and no particular clustering or distribution among different phases (Figure 2a). A detailed examination of PCA on the fecal metabolome data revealed peaks corresponding to carbohydrates toward the PC2 negative direction (Figure 2b, rectangle).

A network analysis of the microbiome and metabolites indicated a relatively large amount of *Bifidobacterium*, fructose, and glucose in the samples (Figure 2c). The network analysis of individuals revealed that some participants showed distinctively increased *Bifidobacterium* and fructose during the FOS phase compared with the pre and post phases (Supplementary Figure S4). The analysis specifically focused on fructose throughout the study period revealed that three participants (i.e., ID04, ID09, and ID11) clearly showed increased fructose in their fecal samples collected in the FOS phase (Figure 2d and Supplementary Figure S4). Results from PCA also confirmed that these three participants had a significantly (*p* < 0.01) distinct amount of fructose compared to the rest of the participants (Figure 2e). In addition to carbohydrates, the presence of other metabolites in fecal samples, including SCFAs, was analyzed throughout the study period (Figure 2f). There were large differences in the metabolites produced throughout the study period among individuals that resulted in no significant differences in these metabolites in fecal samples at different phases.

### 2.3. Genetic Differences in Fructose Metabolism from Metagenomic Analysis

**Figure 3:**
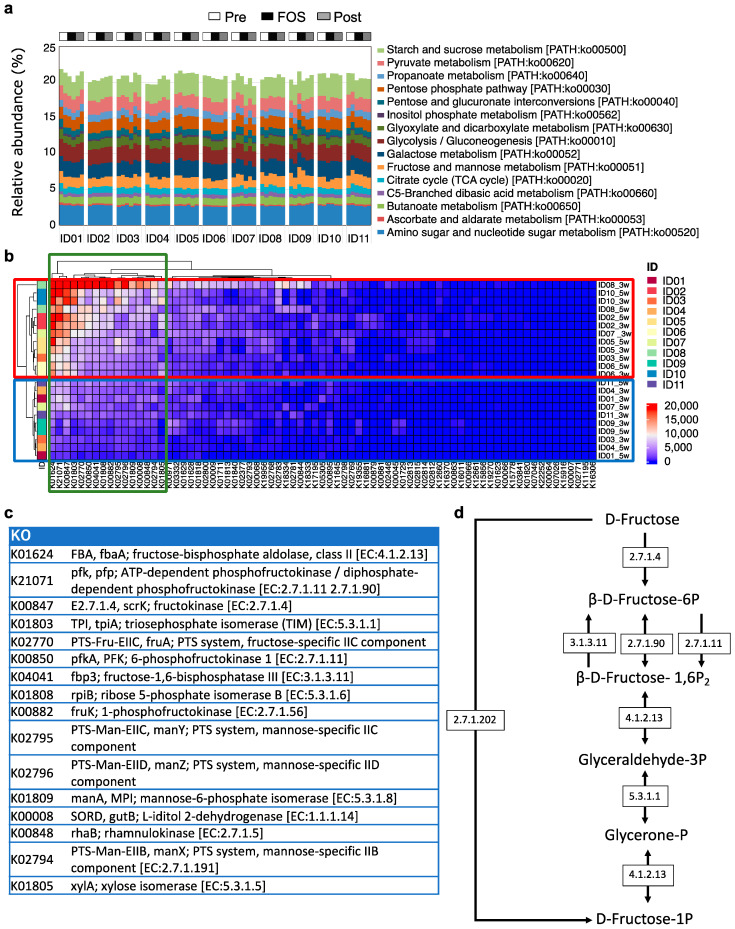
KEGG pathways in carbohydrate metabolism with the metagenomic analysis. (**a**) Relative abundance of the KEGG orthologies (KOs) associated with carbohydrate metabolism in fecal samples was inferred based on the metagenome analysis. (**b**) A comparison of KOs of the fructose and mannose metabolism pathways in individuals obtained from the metagenome analysis of fecal samples using a heatmap calculated by multiplying individual metagenomic genes and a number of bacteria. The green rectangle indicates the gut microbial fructose and mannose metabolism KOs with high variability, and red and blue rectangles indicate groups of individuals having relatively higher and lower expressions of these highly variable KOs, respectively. (**c**) A list of 16 KOs with high individual variability in gene expression that was identified from (**b**). (**d**) Summary of a metabolic pathway that involves many KOs listed in (**c**).

In order to gain mechanistic insights into the individual variation in the amount of fructose in their feces, a metagenomic sequencing that focused on the genetic pathways categorized as carbohydrate metabolism in the Kyoto Encyclopedia of Genes and Genomes (KEGG) pathways was conducted. A relative abundance of genes associated with carbohydrate metabolism showed differences in response to FOS supplementation (Figure 3a). This KEGG analysis showed individual differences in gene expressions were particularly eminent in those of at least 16 carbohydrate metabolic enzymes (Figure 3b, green rectangle), including fructose-bisphosphate aldolase, ATP-dependent phosphofructokinase/diphosphate-dependent phosphofructokinase, and fructokinase (Figure 3b,c). The majority of these enzymes are involved in fructose metabolism (Figure 3d). These analyses indicate that there is individual variability in the fructose metabolism that could result in different fructose consumptions in response to FOS supplementation. The gut microbiome of ID02, ID05, ID06, ID8, and ID10 had a relatively higher possession of genes encoding these enzymes related to fructose metabolism (Figure 3b, red rectangle) which likely results in no accumulation of fructose in their feces collected during the FOS phase, while the gut microbiomes of the other participants (i.e., ID01, ID04, ID09, and ID11) were almost devoid of genes encoding these enzymes, supposedly leading to the higher amounts of fructose in their feces upon FOS supplementation.

### 2.4. Impact of FOS Intake on the Immune Compartment

**Figure 4:**
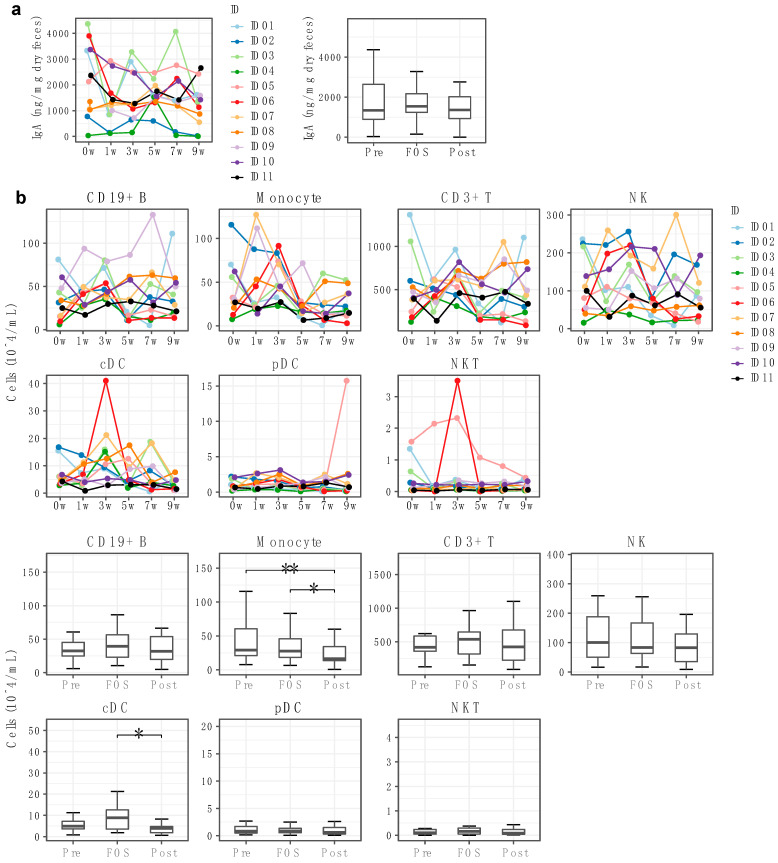
FOS intake and the immune system. (**a**) Fecal IgA at different time points. (**b**) The absolute number of immune cells at each time point. Proportion of immune cells was analyzed using FACS with antibodies listed in Table S2. CD19+ B, B cells; CD3+ T, T cells; NK, natural killer cells; cDC, conventional dendritic cells; pDC, plasmacytoid dendritic cells; NKT, natural killer T cells. * = padj < 0.05, ** = padj < 0.01.

FOS supplementation has been reported to affect the immune system including the augmented secretion of IgA [15,16]. Therefore, we also examined the effect of FOS intake on the immune compartment. There was again a large individual variation and no significant increase in the fecal IgA concentration during FOS intake, although it tended to increase in some individuals (Figure 4a).

Investigation of the composition of PBMCs using FACS also showed individual differences throughout the study period (Figure 4b). While most PBMC subsets did not show significant differences in response to the FOS supplementation, the number of monocytes during the FOS phase was significantly (*p* < 0.01) less than in the pre phase, and the number in the post phase was significantly (*p* < 0.05) less than the FOS period. In addition to monocytes, a number of conventional dendritic cells (pDC) showed a significant (*p* < 0.05) reduction in the post phase compared to the number in the FOS phase. However, the observed results may be partly due to a considerable increase of cDC by one participant (i.e., ID06) during the FOS phase.

## 3. Discussion

It is well documented that microbiota residing in the host vary among individuals [17,21,22,23,24]. In the present study, responses of microbiota and resultant metabolites, as well as the host immune capacity to FOS supplementation, were investigated. While it is common to report changes in the microbiome in relative abundance, the present study focused on absolute numbers of bacteria, as recent studies reported the importance of analyzing microbes using absolute numbers for understanding health risks and observing inter-microbial relationships [25,26,27]. Overall, the study confirmed a high variability of gut microbiomes among individuals, which was consistent with previous studies [17,21,22]. Because of such variability and the small sample size, we were unable to observe the presence of significant changes in the total number as well as the relative abundance of gut bacteria in response to FOS intake as a group. However, when the collected fecal samples were analyzed intra-individually, FOS intake drastically impacted the gut environment and resulted in an increase in *Bifidobacterium*. The observation is comparable to a previous study [13], and, therefore, it can be considered that FOS has a positive impact on the inhabitation of *Bifidobacterium* in the gut. Interestingly, although there was no significant difference in the absolute number of *Blautia* throughout the study period, we observed a small but significant reduction in its relative abundance during FOS intake. This suggests that while *Bifidobacterium* increased both the actual number and the proportion relative to the total number of bacteria, *Blautia* did not change its number but reduced its proportion during the FOS supplementation period. The results also suggest that the FOS supplementation decreases microbial diversity in fecal samples as confirmed by the Shannon index, probably due to the large increase of *Bifidobacterium*.

Loss of bacterial diversity in feces has been reported to be associated with disorders such as inflammatory bowel disease [6,28,29,30]. However, to the best of our knowledge, there is no report of the influence of dietary components on gut microbial diversity. Therefore, the observed reduction of gut microbial diversity by FOS supplementation is surprising. Since the intervention period in the present study was relatively short (i.e., four weeks), further investigation into the long-term effects, as well as the associated physiological significance, is warranted.

It Is hypothesized that supplemented FOS should be easily digested by gut microbes and utilized to produce microbial metabolites such as SCFAs. Hence, participants were assumed to show an increase in carbohydrates, especially an amount of fructose during the FOS supplementation period. However, the present study revealed considerable individual variability in the amount of fructose produced during the FOS phase and only three participants (ID04, ID09, and ID11) showed a significant increase of fructose in fecal samples collected during the FOS phase. Further analysis using a metagenomic approach showed that all three participants showed marginal gene expressions of the enzymes involved in the fructose metabolism pathway, including fructose-bisphosphate aldolase, fructokinase, and fructose-1,6-bisphosphatase III. Therefore, these three participants may be considered individuals with poor metabolic capacity in fructose catabolism. This suggests that FOS supplementation does not provide the same degree of health benefits to every individual, and caution is required for FOS supplementation. In fact, fructose consumption is linked to the rising incidence of obesity and cancer, which are two of the leading causes of morbidity and mortality globally [31,32]. Although monosaccharides including fructose are generally thought to be absorbed in the small intestine, fructose could be possibly absorbed in the colon as well since a fructose transporter GLUT7 is also expressed in the colon [33,34]. In this regard, FOS administration should be cautiously administered to individuals whose gut microbiota is insufficient in fructose catabolism. In the present study, ID01 also showed low gene expression of fructose metabolism-related enzymes but did not show a significant increase in fructose during the FOS supplementation period. Therefore, it should be considered that other metabolic pathways may also be involved in fructose degradation.

Previous studies reported that at least 21 species in the gut can metabolize FOS [35], including lactic acid-producing bacteria such as *Bifidobacterium* and Lactobacillus [12,13]. Therefore, the total number and relative abundance of these species within microbiota may affect the efficiency of FOS degradation. In addition, it has been reported that a strain of *Bifidobacterium* with specific ATP-binding cassette (ABC) carbohydrate transporters [20] can contribute to the efficiency of the clearance of fructose.

In the present study, no clear changes in metabolites were observed. This is likely due to large inter-individual variability in the gut microbiome and the efficiency of FOS degradation. Fermentation of FOS by *Bifidobacterium* is known to produce acetate and lactate and is further utilized by other gut microbes such as *Eubacterium* and *Roseburia* to produce butyrate [36,37]. In addition, *Bacteroides*, *Faecalibacterium*, *Clostridium*, and *Ruminococcus*, may increase the production of lactate, propionate, and butylate [21,37,38]. Although FOS supplementation increased the total number and a relative abundance of *Bifidbacterium*, we were unable to observe a clear pattern of increase in *Bifidobacterium*-related metabolites (e.g., acetate and lactate) and the reduction of other metabolites upon FOS intake. Considering the presence of individuals with efficient fructose metabolism, it may be possible to investigate the effects of FOS intake by a careful selection of participants with influencing factors (e.g., an absolute and relative abundance of *Bifidobacterium*, a presence of *Bifidobacterium* strains with ABC fructose transporters, and a low expression of enzymes involved in the fructose metabolism pathway).

Consumption of FOS has been reported to have beneficial effects on the immune functions of the host [14,15,16]. Due to the large inter-individual variability of the gut microbiome and small sample size, the present study was unable to indicate statistical significance for changes in immune functions from FOS supplementation. Nevertheless, the fecal samples showed a tendency for increasing IgA in response to FOS intake. Previous studies have reported the increase in fecal IgA upon FOS intake in experimental animals such as mice and rats [39]. By contrast, fecal IgA tended to increase, but there was no statistical significance from FOS intake in human studies [17,39], consistent with the present study. This discrepancy could at least partly be because human studies reflect large inter-individual differences in factors such as genetic backgrounds and diets, whereas these factors are much more uniform in experimental animals. In addition to IgA, FACS analysis of PMBC showed a significant decrease in monocytes and an increase in cDC during FOS supplementation. This may suggest that short-term supplementation of FOS could cause an acute change in the systemic immune status of these cells regardless of differences in the gut microbiome.

## 4. Materials and Methods

### 4.1. Participants

Eleven Japanese males aged between 30 and 50 years were recruited. Participants were excluded if they were diagnosed or susceptible to immune dysfunctions including human immunodeficiency virus (HIV) infection; had undergone any operations on the gastrointestinal tract excluding the gallbladder and appendix in the last five years; had any prescription of medications, or were under treatment on the gastrointestinal tract; had chronic constipation; or were under treatment or susceptible to toxic shock syndrome. In order to remove the influence of gender on the composition of the gut microbiome and its response to FOS supplementation, females were also excluded from the study. All participants were given information documents and a verbal explanation about the study and written informed consent was obtained prior to their participation in the study. Ethical approval was obtained from the human research ethics committee of relevant institutions prior to the commencement of the study, and all methods were performed in accordance with the relevant guidelines and regulations. Participants’ self-reported age, height, weight, and body mass index (BMI) were 37.4 ± 4.6 years (range: 30.0–44.0 years), 172.7 ± 6.0 cm (range: 159.0–180.0 cm), 75.4 ± 15.4 kg (range: 58.0–110.0 kg), and 25.2 ± 4.5 kg/m^2^ (range: 20.1–33.9 kg/m^2^), respectively (Supplementary Table S1).

### 4.2. Study Design

The study was conducted as a nine-week longitudinal study. After two weeks of the baseline period (the “Pre” phase), participants were instructed to consume FOS supplements as described below for four weeks (the “FOS” phase). After this intervention period, changes in the participants’ gut microbiome and its metabolites were monitored for three weeks (the “Post” phase) (Supplementary Figure S1). Participants were instructed to maintain an ordinary lifestyle during the entire period of the study.

### 4.3. FOS Supplementation

Participants were instructed to consume 12 g of a FOS syrup (MeioligoP^®^, Meiji Food Materia Co., Ltd., Tokyo, Japan) for 28 consecutive days. The syrup consists of more than 95% FOS with less than 5% glucose, fructose, and sucrose. The syrup was prepared into two packages each containing 6 g of FOS. It was recommended to participants to consume each package at different times of the day in order to prevent the possibility of having a loose stool.

### 4.4. Sample Collection

Samples of blood and feces were collected at the Pre-, FOS, and Post-phases of the study. Two samples were collected at each phase, and, therefore, a total of six samples were collected (Supplementary Figure S1). Blood samples were collected from the median cubital vein by medical personnel. A total of 5 mL of peripheral blood was collected into a blood collection tube containing EDTA-2Na (Venoject II VP-NA050K, Terumo Corp., Tokyo, Japan). Fecal samples were also collected using a stool collection kit consisting of collection sheets, plastic spoons, and plastic containers. Participants were instructed to wear sterile gloves and collect about 3 g of feces without any contamination of toilet water or urine within 24 h prior to blood sampling, and the samples were stored in a fridge. Samples submitted to the research group were immediately stored in a freezer at −80 °C until analysis.

### 4.5. Measurement of Fecal IgA

Fecal samples were lyophilized using a VD-800R lyophilizer (TAITEC Co., Ltd., Saitama, Japan) for 24 h. Freeze-dried feces were then ground with 3.0 mm Zirconia Beads (BioSpec Products, OK, USA) using a Shake Master (Biomedical Science Corp., Tokyo, Japan) for 10 min. Approximately 10 mg of each fecal sample was suspended in 300 μL of phosphate-buffered saline (PBS) containing proteinase inhibitor cocktail tablets (Complete EDTA-free, Roche Diagnostics GmbH, Mannheim, Germany) and homogenized for 1 min. After being centrifuged at 17,800× *g* for 15 min at 4 °C, the supernatant was collected from the homogenate sample and frozen at −20 °C. The measurement of fecal IgA was performed according to the instruction of the ELISA kit (E80-102, Bethyl Laboratories Inc., Montgomery, TX, USA). 

### 4.6. Bacterial DNA Extraction

DNA extraction from feces was performed according to the literature with minor modifications [40,41]. Bacterial pellets from feces were suspended with a 10 mM Tris-HCl/10 mM EDTA solution. The sample suspension was incubated with 15 mg/mL lysozyme (FUJIFILM Wako Pure Chemical Corp., Osaka, Japan) at 37 °C for 1 h. A final concentration of 2000 units/mL of purified achromopeptidase (FUJIFILM Wako Pure Chemical Corp., Osaka, Japan) was added and then incubated at 37 °C for 30 min. The suspension was added at 1% (*w/v*) sodium dodecyl sulfate and 1 mg/mL proteinase K (Merck KGaA) and incubated at 55 °C for 1 h. After centrifugation, the bacterial DNA was purified using a phenol/chloroform/isoamyl alcohol (25:24:1) solution. The DNA was precipitated by adding ethanol and sodium acetate, and RNase treatment and polyethylene glycol (PEG) precipitation were performed.

### 4.7. Bacterial 16S rRNA Gene Amplicon Sequencing and Processing

The 16S rRNA gene V1-V2 regions were amplified by PCR with barcoded bacterial universal primers (27Fmod and 338R) [40]. Amplification of V1-V2 regions and preparation of the sequencing library were performed according to the literature with minor modifications [42,43]. Equal amounts of multiplexed PCR amplicon were mixed and then sequenced using either 454 GS FLX Titanium or 454 GS JUNIOR (Roche Diagnostics GmbH, Mannheim, Germany).

Analysis of the 16S rRNA gene sequencing data was conducted using an analysis pipeline established by the research group. Each sample was demultiplexed based on sample-specific barcodes followed by the removal of reads lacking both forward and reverse primer sequences. The sequence data were denoised by removing sequences with an average quality of <25 and possible chimeric sequences. We performed chimera checking and taxonomy assignment using the database constructed from public databases (Ribosomal Database Project (RDP) v. 10.27, CORE (http://microbiome.osu.edu/, accessed on 1 April 2020) and a reference genome sequence database obtained from the NCBI FTP site (ftp://ftp.ncbi.nih.gov/genbank/, accessed on 1 December 2011)). Reads removed in these processes accounted for about 64.6% of all reads, most of which represented reads lacking PCR primer sequences (63.4% of raw reads). Three thousand reads per sample were randomly selected from each filter-passed read and were used for further analysis since this read number represents more than 90% of the total reads according to Good’s coverage estimator [44]. These high-quality reads were clustered into operational taxonomic units (OTUs) using a 96% pairwise-identity using UCLUST (http://www.drive5.com/, accessed on 29 August 2022). Taxonomic assignments of OTUs were performed against the database using the GLsearch program accessed on 29 August 2022. The weighted UniFrac distance [45] was calculated to assess the distance between each sample. Shannon’s diversity index (Shannon Index) was calculated to evaluate the diversity of microbial communities in a sample using the vegan package of R software (version 3.6.1). All of the 16S rRNA sequence data used in this study were deposited in DDBJ/GenBank/EMBL under accession numbers: DRA014786.

### 4.8. Metagenomic Sequencing

Metagenome shotgun libraries (insert size of ~500 bp) were prepared using the TruSeq Nano DNA kit (Illumina) and sequenced by the Illumina NovaSeq platform. After quality filtering, reads mapped to the human genome (HG19) and the phiX bacteriophage genome were removed.

### 4.9. Assembly of Metagenomic Sequences and Gene Prediction

For each individual, the filter-passed NovaSeq reads were assembled using MEGAHIT (v1.2.4). Prodigal (v2.6.3) was used to predict protein-coding genes (≥100 bp) in the contigs (≥500 bp) and singletons (≥300 bp). Finally, 2,234,201 non-redundant genes were identified in the 290 samples by clustering the predicted genes using CD-HIT with a 95% nucleotide identity and 90% length coverage cut-off.

### 4.10. Functional Assignment of Non-Redundant Genes in Human Gut Microbiomes

Functional assignment of the non-redundant genes was performed using DIAMOND (*e*-value ≤ 1.0 × 10^−5^) against the Kyoto Encyclopedia of Genes and Genomes (KEGG) database (release 7 October 2019) to obtain the KEGG orthologies (KOs). The genes with the best hit correlating to eukaryotic genes were excluded from further analysis.

### 4.11. Quantification of the Annotated Genes in Human Gut Microbiomes

A million metagenomic reads per individual were mapped to the JPGM [7] and IGC [46] merged reference gene set using Bowtie2 with a 95% identity cut-off. The number of reads that mapped equally to more than one gene was normalized by the proportion of the number of reads uniquely mapped to the genes as was conducted for the mapping analysis to the reference genomes. The proportion of KOs was calculated from the number of reads mapped to them.

### 4.12. Metabolic Profiling of Human Fecal Samples

Lyophilized fecal samples were extracted in a 100 mmol/L potassium phosphate buffer (in deuterium oxide containing 1 mmol/L sodium 2,2-dimethyl-2-silapentane-5-sulfonate, pH = 7.0) as previously described [47]. In total, 0.8 mL of the above samples was inserted into a 5 mmϕ-Nuclear Magnetic Resonance (NMR) tube, then NMR experiments were conducted using a 700 MHz- NMR spectrometer (Bruker AVANCE II 700, Bruker Biospin GmbH, Rheinstetten, Germany). All ^1^H NMR spectra were acquired using a Bruker standard pulse program “p3919gp” with 32 K data points, 32 scans, 16 dummy scans, 14 ppm spectral width, and 3 s relaxation delay [48]. For the annotation of the signals detected in the ^1^H NMR spectra, two-dimensional J-resolved NMR measurements were performed using a Bruker standard pulse program “jresgpprqf” with 32 data points for F1 and 16 K data points for F2, 8 scans, 16 dummy scans, a 50 Hz spectral width for F1 and 16 ppm spectral width for F2, and 1.5 s relaxation delay, as described previously [49]. The detected signals were annotated using the SpinCouple program (http://dmar.riken.jp/spincouple/, accessed on 29 August 2022) [50] with reference to the Human Metabolome Database (http://www.hmdb.ca/, accessed on 29 August 2022) [51].

### 4.13. Quantification of Bacterial Number and Gene Expression Using Quantitative Polymerase Chain Reaction (qPCR)

The universal 16S rRNA primers (27Fmod: 5′-AGRGTTTGATYMTGGCTCAG-3′ and 338R: 5′-TGCTGCCTCCCGTAGGAGT-3′) were used to estimate the microbial cell number by real-time qPCR. DNA of *Escherichia coli* that had been counted by the Colony Forming Unit (CFU) assay was used as a standard. Fecal DNA was assayed in 20 μL PCR reactions according to the protocol for TB Green Premix Ex Taq II (Tli RNaseH Plus) (TaKaRa Bio, Tokyo, Japan). Each reaction mixture contained 10 pmol of each primer, 10 µL of TB Green Premix Ex Taq II (Tli RNase H Plus) (Takara Bio, Tokyo, Japan), extracted DNA, and UltraPure DNase/RNase-Free Distilled Water (Thermo Fisher Scientific, Waltham, MA, USA) to reach a final volume of 20 μL. PCR conditions were as follows: 95 °C for 30 s and then 40 cycles of 95 °C for 5 s and 60 °C for 30 s. The threshold cycle (Ct) value of qPCR was calculated by the 2nd Derivative Maximum (SDM) method using the Thermal Cycler Dice Real Time System TP800 (Takara Bio, Tokyo, Japan). To estimate the absolute abundance of bacteria in the fecal sample, the log10-fold standard curves ranging from 10^4^ to 10^9^ copies were produced using the DNA of *E. coli*. The threshold cycle values were converted into the estimates of the absolute abundance of bacteria in the fecal samples (copy numbers/g of dry feces).

### 4.14. Correlation Network Construction

A correlation network was built from the relative intensities of metabolites assigned by metabolome data measured by NMR, and the relative abundance of the microbiome at the genus level by 16S rRNA amplicon sequencing. Edges of the network show significant pairwise interactions between measurements using Spearman’s rank correlation coefficient. Nodes’ sizes represent the proportion to relative scores computed for each measurement. The two-dimensional correlation network was drawn by the Cytoscape software v3.9.1 [52].

### 4.15. Peripheral Blood Mononuclear Cell (PBMC) Isolation and Analysis

PBMCs were isolated by Ficoll-Paque (GE Healthcare Life Sciences, Tokyo, Japan) density gradient centrifugation and suspended in 2% FCS/D-PBS. Specific volumes of antibodies that are listed in Supplementary Table S3 were added to 50 μL of the cell suspensions for 30 min at 4 °C. In total, 1 mL of 2% FCS/D-PBS was added to the stained cell suspensions, centrifuged at 1200 rpm for 5 min at 4 °C, washed with 2% FCS/D-PBS twice, resuspended in 300 μL of 2% FCS/D-PBS, and analyzed using a FACS Canto II (BD Bioscience, Franklin Lakes, NJ, USA). The gating strategy is shown in Supplementary Figure S2. A fraction of cells within each gating scheme was used for subsequent analysis.

### 4.16. Multivariate Statistical Analysis

Statistical analyses were conducted with R (version 3.6.1). Depending on the normality of the data, Mann–Whitney’s U-test or Kruskal–Wallis test was used to perform statistical analysis among a comparison of the 3 groups. Adonis was performed for the principal coordinates analysis (PCoA) of microbiomes and the changes in fructose concentration over time in feces from the individuals. For time-course analysis of SCFAs, repeated one-way ANOVA was applied. The *p*-values were corrected for multiple testing using the Benjamini-Hochberg (BH) method.

## 5. Conclusions

The present study confirmed large inter-individual variability in the gut ecosystem and its response to FOS supplementation. This study also suggests that differences in the gene expression of enzymes that are involved in the fructose metabolism pathway contribute to individual differences in fructose catabolism upon their FOS intake. Furthermore, the study shows an acute response in the immune system regardless of individual variability in the composition of the gut microbiome. Since the study was conducted with only 11 male participants, the results cannot be generalized, and further studies are necessary with a larger sample size, and the inclusion of females in future research, to obtain more comprehensive conclusions. Nevertheless, the present study provided insight into the potential factors that influence the effects of FOS supplementation and emphasizes the importance of understanding individual variability. The accumulation of “personalized” knowledge on gut ecosystems may enable effective instructions on prebiotic consumption to optimize health benefits for individuals in the future.

## Data Availability

All sequence data are available from DDBJ DRA under DRA014786.

## Supplementary Materials

The following supporting information can be downloaded at: https://www.mdpi.com/article/10.3390/ijms231911728/s1.
