# Supplementary material for: Integrated Multi-Omics Analysis Reveals Differential Effects of Fructo-Oligosaccharides (FOS) Supplementation on the Human Gut Ecosystem
Source: Int J Mol Sci. 2022 Oct 3;23(19):11728. doi: 10.3390/ijms231911728 (PMC9569659; doi:10.3390/ijms231911728)
Supplement: Supplementary file 1 [file ijms-23-11728-s001.zip › Supplementary_information.pptx]

## Slide 1
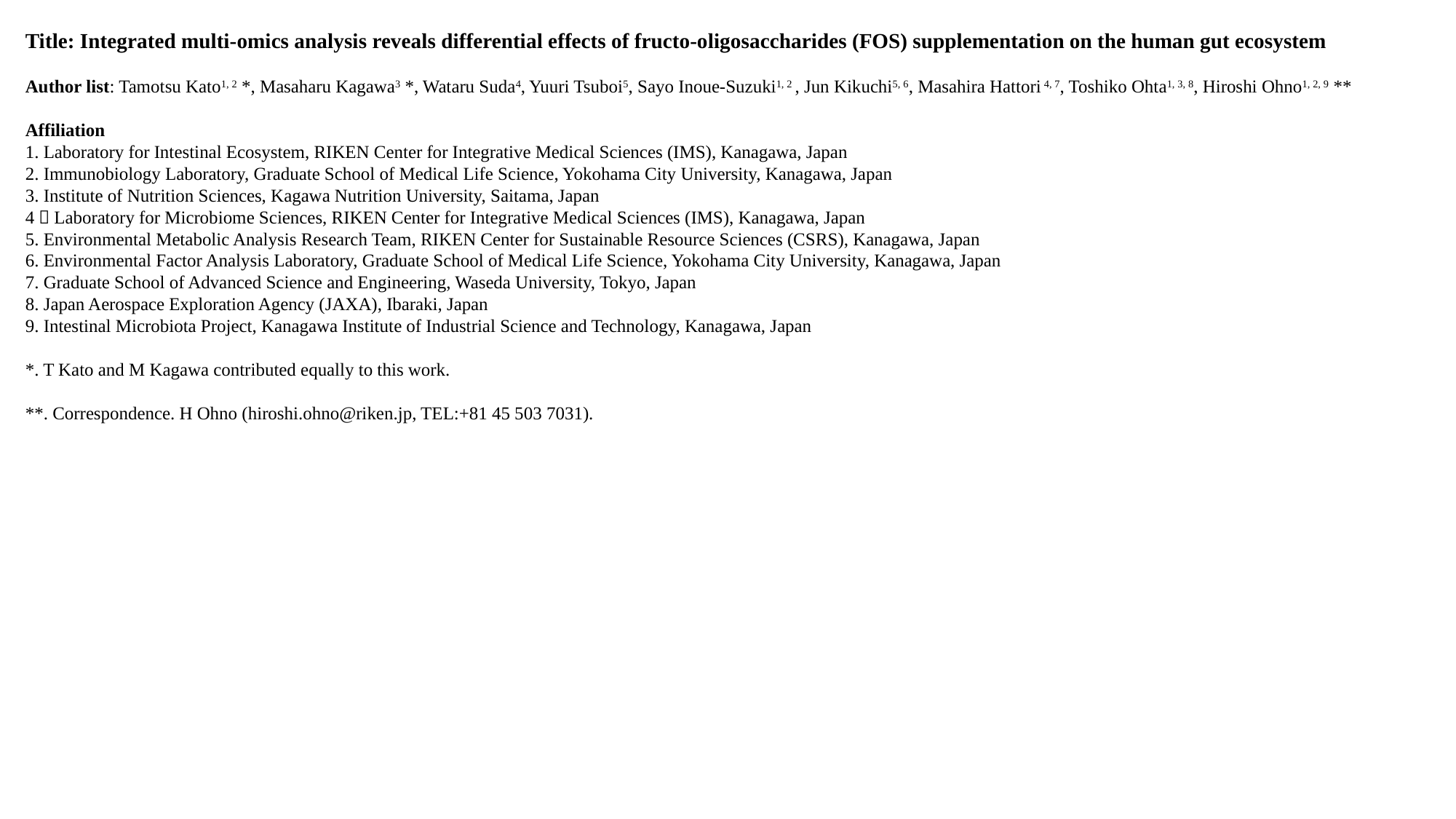

Title: Integrated multi-omics analysis reveals differential effects of fructo-oligosaccharides (FOS) supplementation on the human gut ecosystem
Author list: Tamotsu Kato1, 2 *, Masaharu Kagawa3 *, Wataru Suda4, Yuuri Tsuboi5, Sayo Inoue-Suzuki1, 2 , Jun Kikuchi5, 6, Masahira Hattori 4, 7, Toshiko Ohta1, 3, 8, Hiroshi Ohno1, 2, 9 **
Affiliation
1. Laboratory for Intestinal Ecosystem, RIKEN Center for Integrative Medical Sciences (IMS), Kanagawa, Japan
2. Immunobiology Laboratory, Graduate School of Medical Life Science, Yokohama City University, Kanagawa, Japan
3. Institute of Nutrition Sciences, Kagawa Nutrition University, Saitama, Japan
4．Laboratory for Microbiome Sciences, RIKEN Center for Integrative Medical Sciences (IMS), Kanagawa, Japan
5. Environmental Metabolic Analysis Research Team, RIKEN Center for Sustainable Resource Sciences (CSRS), Kanagawa, Japan
6. Environmental Factor Analysis Laboratory, Graduate School of Medical Life Science, Yokohama City University, Kanagawa, Japan
7. Graduate School of Advanced Science and Engineering, Waseda University, Tokyo, Japan
8. Japan Aerospace Exploration Agency (JAXA), Ibaraki, Japan
9. Intestinal Microbiota Project, Kanagawa Institute of Industrial Science and Technology, Kanagawa, Japan
*. T Kato and M Kagawa contributed equally to this work.
**. Correspondence. H Ohno (hiroshi.ohno@riken.jp, TEL:+81 45 503 7031).

## Slide 2
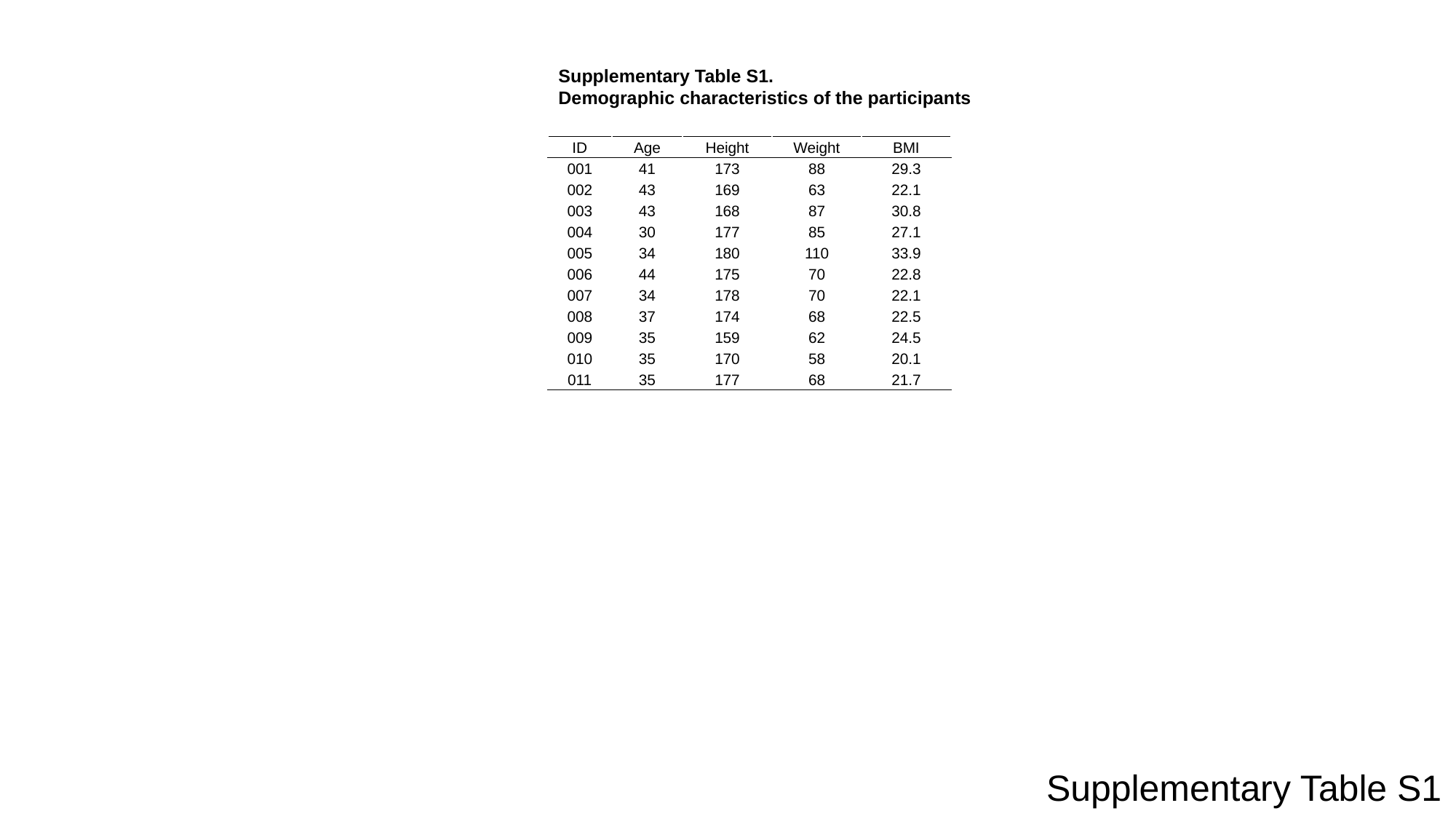

Supplementary Table S1.
Demographic characteristics of the participants
| ID | Age | Height | Weight | BMI |
| --- | --- | --- | --- | --- |
| 001 | 41 | 173 | 88 | 29.3 |
| 002 | 43 | 169 | 63 | 22.1 |
| 003 | 43 | 168 | 87 | 30.8 |
| 004 | 30 | 177 | 85 | 27.1 |
| 005 | 34 | 180 | 110 | 33.9 |
| 006 | 44 | 175 | 70 | 22.8 |
| 007 | 34 | 178 | 70 | 22.1 |
| 008 | 37 | 174 | 68 | 22.5 |
| 009 | 35 | 159 | 62 | 24.5 |
| 010 | 35 | 170 | 58 | 20.1 |
| 011 | 35 | 177 | 68 | 21.7 |
Supplementary Table S1

## Slide 3
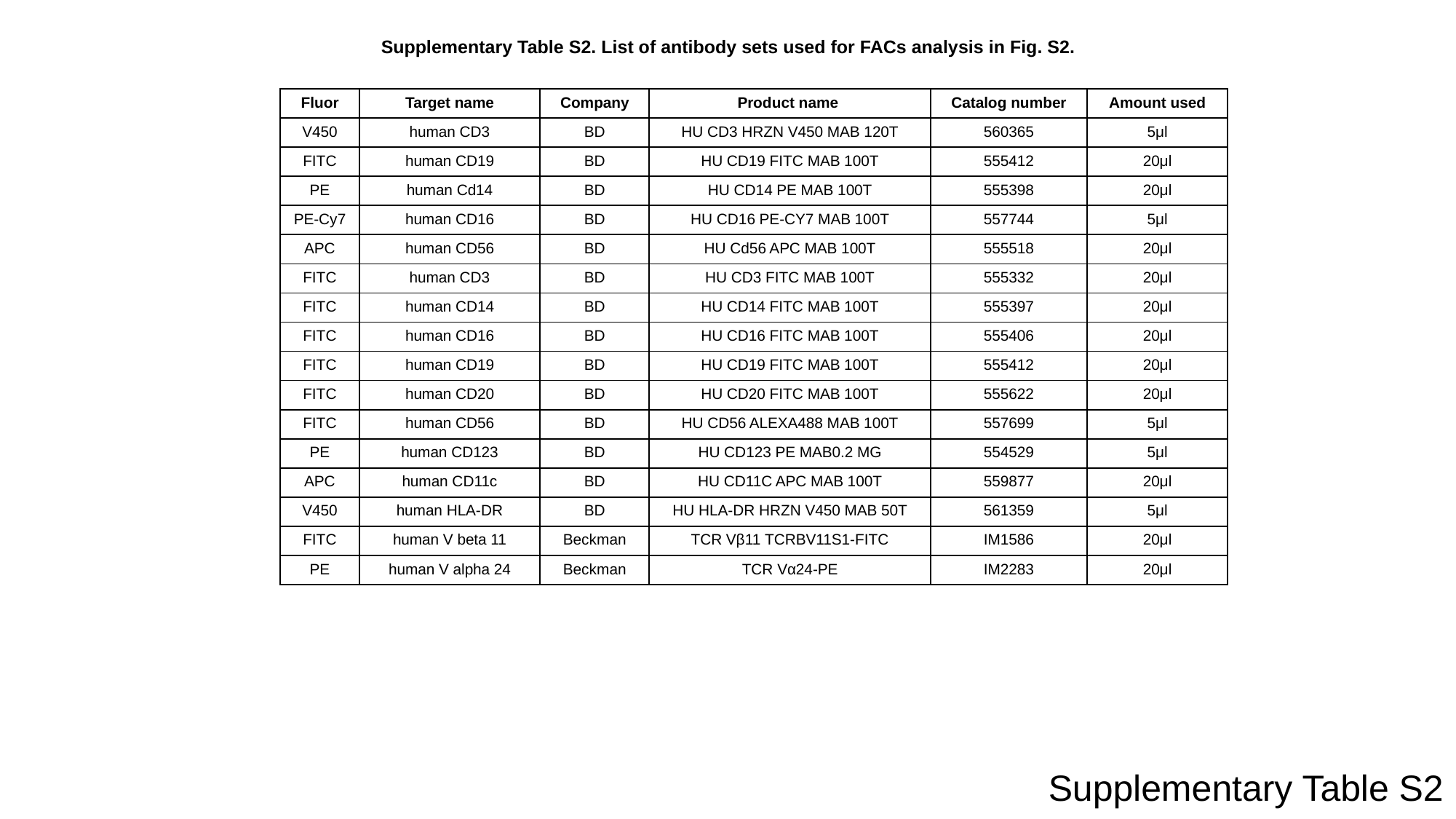

Supplementary Table S2. List of antibody sets used for FACs analysis in Fig. S2.
| Fluor | Target name | Company | Product name | Catalog number | Amount used |
| --- | --- | --- | --- | --- | --- |
| V450 | human CD3 | BD | HU CD3 HRZN V450 MAB 120T | 560365 | 5μl |
| FITC | human CD19 | BD | HU CD19 FITC MAB 100T | 555412 | 20μl |
| PE | human Cd14 | BD | HU CD14 PE MAB 100T | 555398 | 20μl |
| PE-Cy7 | human CD16 | BD | HU CD16 PE-CY7 MAB 100T | 557744 | 5μl |
| APC | human CD56 | BD | HU Cd56 APC MAB 100T | 555518 | 20μl |
| FITC | human CD3 | BD | HU CD3 FITC MAB 100T | 555332 | 20μl |
| FITC | human CD14 | BD | HU CD14 FITC MAB 100T | 555397 | 20μl |
| FITC | human CD16 | BD | HU CD16 FITC MAB 100T | 555406 | 20μl |
| FITC | human CD19 | BD | HU CD19 FITC MAB 100T | 555412 | 20μl |
| FITC | human CD20 | BD | HU CD20 FITC MAB 100T | 555622 | 20μl |
| FITC | human CD56 | BD | HU CD56 ALEXA488 MAB 100T | 557699 | 5μl |
| PE | human CD123 | BD | HU CD123 PE MAB0.2 MG | 554529 | 5μl |
| APC | human CD11c | BD | HU CD11C APC MAB 100T | 559877 | 20μl |
| V450 | human HLA-DR | BD | HU HLA-DR HRZN V450 MAB 50T | 561359 | 5μl |
| FITC | human V beta 11 | Beckman | TCR Vβ11 TCRBV11S1-FITC | IM1586 | 20μl |
| PE | human V alpha 24 | Beckman | TCR Vα24-PE | IM2283 | 20μl |
Supplementary Table S2

## Slide 4
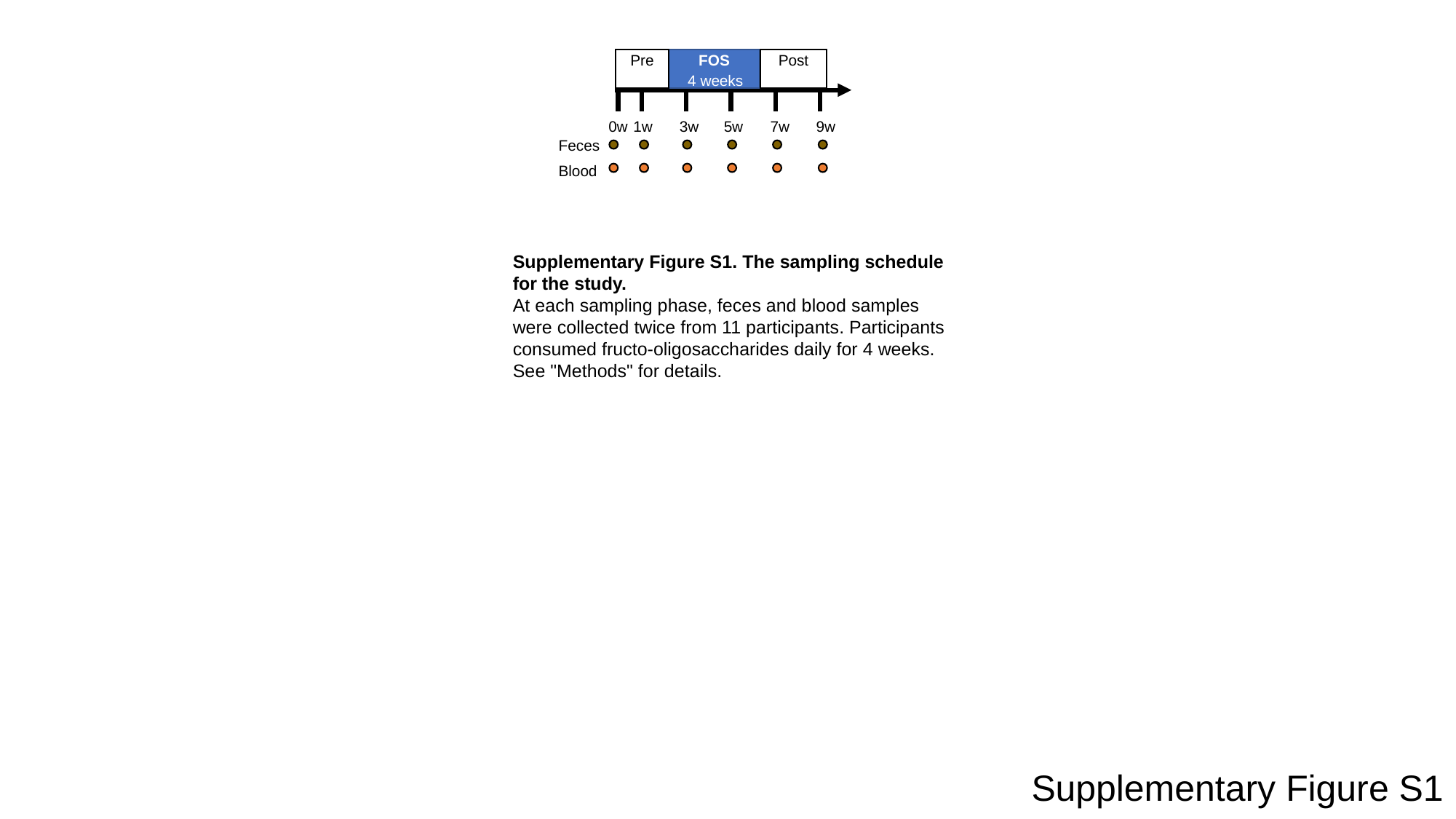

Pre
FOS
Post
4 weeks
9w
0w
1w
3w
5w
7w
Feces
Blood
Supplementary Figure S1. The sampling schedule for the study.
At each sampling phase, feces and blood samples were collected twice from 11 participants. Participants consumed fructo-oligosaccharides daily for 4 weeks.
See "Methods" for details.
Supplementary Figure S1

## Slide 5
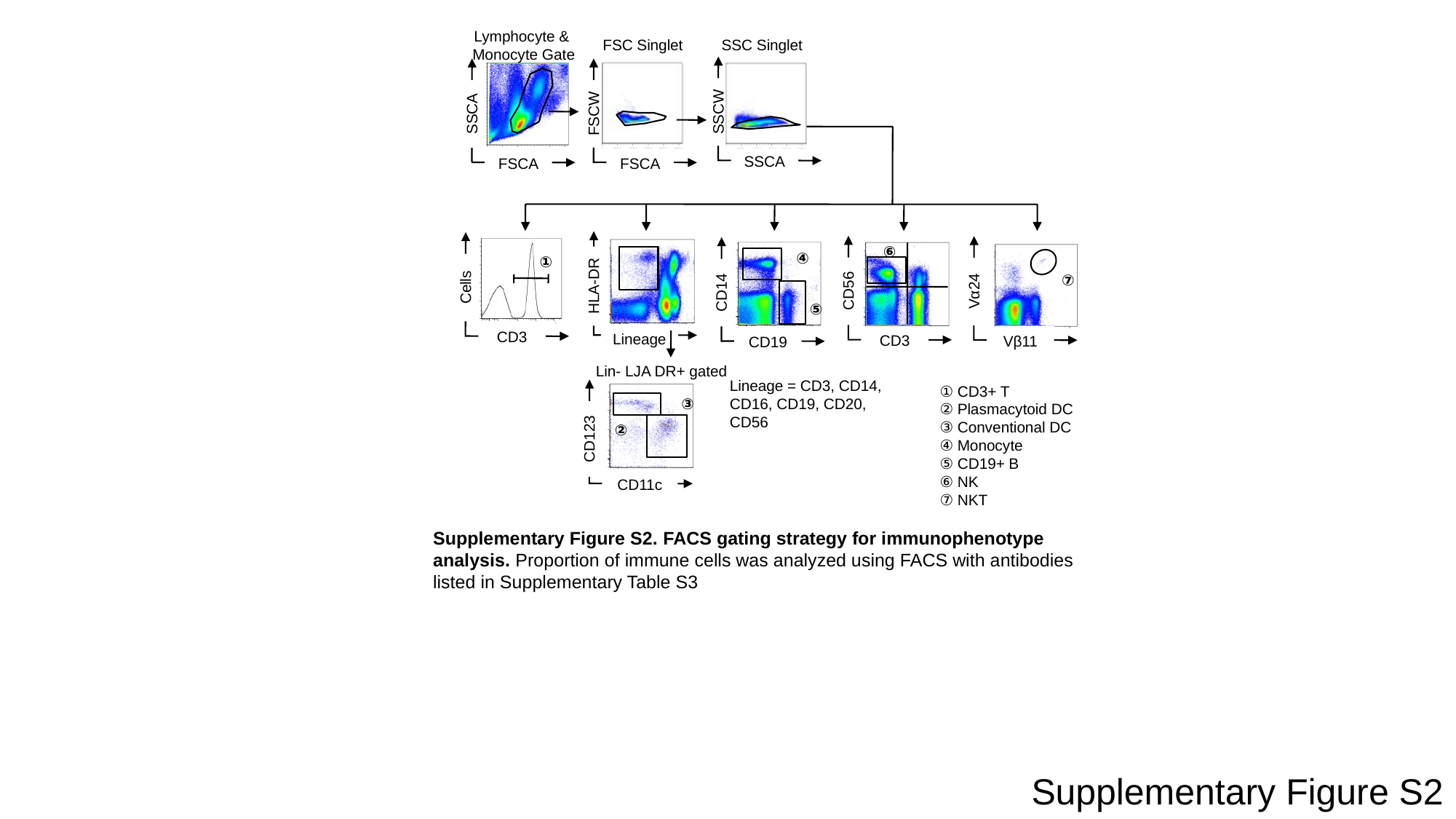

Lymphocyte &
Monocyte Gate
FSC Singlet
SSC Singlet
SSCW
FSCW
SSCA
SSCA
FSCA
FSCA
⑥
④
①
⑦
HLA-DR
Cells
CD56
Vα24
CD14
⑤
CD3
Lineage
CD3
Vβ11
CD19
Lin- LJA DR+ gated
Lineage = CD3, CD14,
CD16, CD19, CD20, CD56
① CD3+ T
② Plasmacytoid DC
③ Conventional DC
④ Monocyte
⑤ CD19+ B
⑥ NK
⑦ NKT
③
②
CD123
CD11c
Supplementary Figure S2. FACS gating strategy for immunophenotype analysis. Proportion of immune cells was analyzed using FACS with antibodies listed in Supplementary Table S3
Supplementary Figure S2

## Slide 6
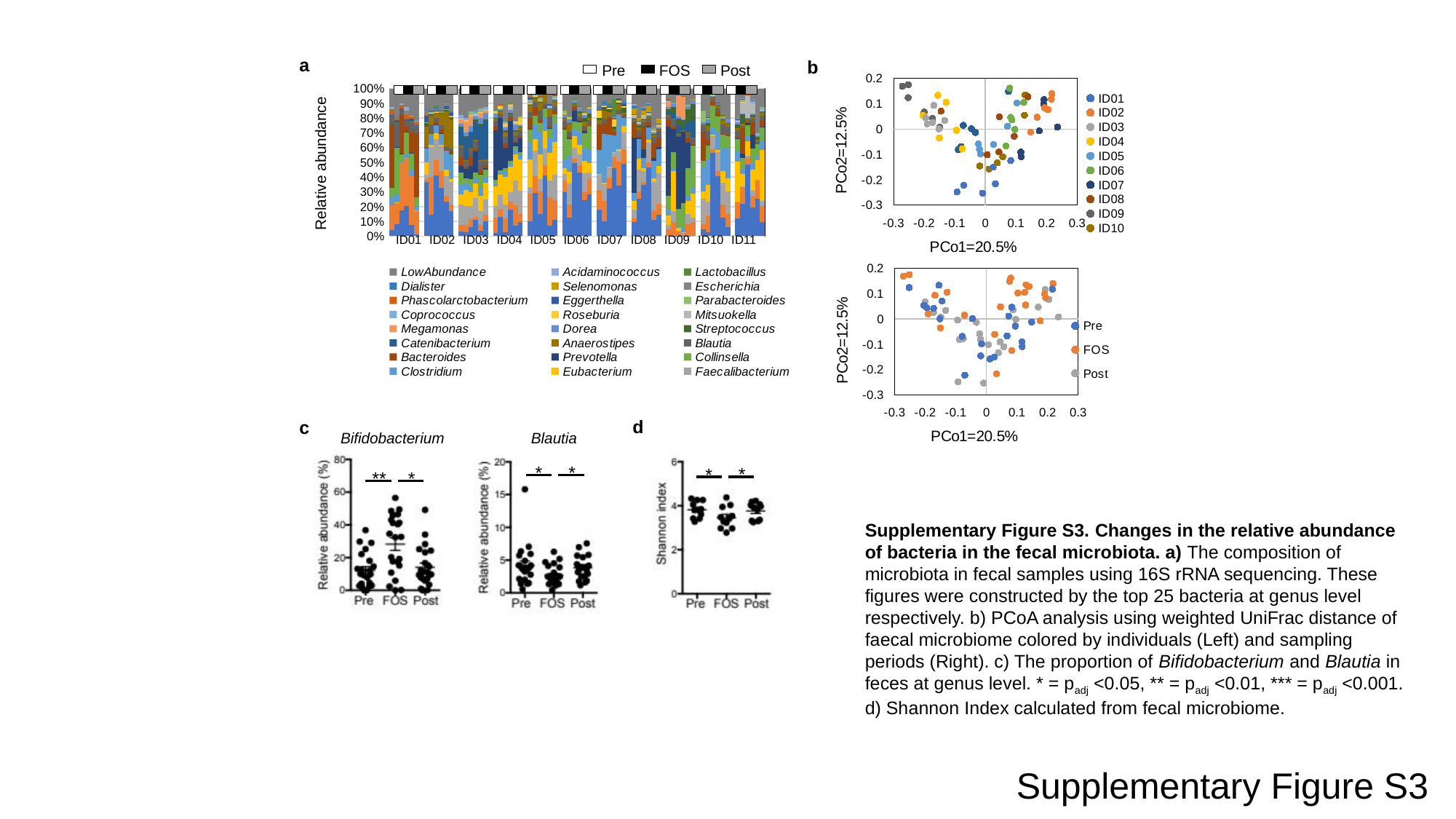

a
b
Pre
FOS
Post
### Chart
| Category | | | | | | | | | | | |
|---|---|---|---|---|---|---|---|---|---|---|---|
### Chart
| Category | Bifidobacterium | Ruminococcus | Faecalibacterium | Eubacterium | Clostridium | Collinsella | Prevotella | Bacteroides | Blautia | Anaerostipes | Catenibacterium | Streptococcus | Dorea | Megamonas | Mitsuokella | Roseburia | Coprococcus | Parabacteroides | Eggerthella | Phascolarctobacterium | Escherichia | Selenomonas | Dialister | Lactobacillus | Acidaminococcus | LowAbundance |
|---|---|---|---|---|---|---|---|---|---|---|---|---|---|---|---|---|---|---|---|---|---|---|---|---|---|---|
| Pre | 0.04066666666666666 | 0.16966666666666666 | 0.0 | 0.0 | 0.04833333333333333 | 0.06733333333333333 | 0.0 | 0.432 | 0.059333333333333335 | 0.001 | 0.0 | 0.0 | 0.0 | 0.0 | 0.0 | 0.0 | 0.0 | 0.0 | 0.0026666666666666666 | 0.035 | 0.0023333333333333335 | 0.0 | 0.0 | 0.0003333333333333333 | 0.018 | 0.1233333333333333 |
| Pre | 0.079 | 0.148 | 0.0 | 0.0003333333333333333 | 0.049 | 0.32666666666666666 | 0.0006666666666666666 | 0.0023333333333333335 | 0.158 | 0.004 | 0.0 | 0.005666666666666667 | 0.0 | 0.0 | 0.0 | 0.0 | 0.0 | 0.0 | 0.013 | 0.013333333333333334 | 0.003 | 0.0 | 0.0 | 0.0006666666666666666 | 0.0 | 0.19633333333333325 |
| FOS | 0.174 | 0.328 | 0.0 | 0.0 | 0.006666666666666667 | 0.050333333333333334 | 0.0006666666666666666 | 0.263 | 0.04133333333333333 | 0.0006666666666666666 | 0.0 | 0.0 | 0.0 | 0.0 | 0.0 | 0.0 | 0.0 | 0.0 | 0.001 | 0.020666666666666667 | 0.004333333333333333 | 0.0 | 0.0 | 0.0 | 0.007666666666666666 | 0.10166666666666657 |
| FOS | 0.203 | 0.32366666666666666 | 0.0003333333333333333 | 0.0 | 0.028 | 0.15366666666666667 | 0.0003333333333333333 | 0.035333333333333335 | 0.039 | 0.0 | 0.0 | 0.005 | 0.0 | 0.0 | 0.0 | 0.0 | 0.0 | 0.0 | 0.0006666666666666666 | 0.032 | 0.030666666666666665 | 0.0 | 0.0 | 0.0 | 0.024666666666666667 | 0.12366666666666681 |
| Post | 0.07766666666666666 | 0.33166666666666667 | 0.0 | 0.0 | 0.035 | 0.12133333333333333 | 0.0003333333333333333 | 0.135 | 0.07566666666666666 | 0.0016666666666666668 | 0.0 | 0.0006666666666666666 | 0.0 | 0.0 | 0.0 | 0.0 | 0.0 | 0.0 | 0.014333333333333333 | 0.017333333333333333 | 0.0026666666666666666 | 0.0 | 0.0 | 0.0 | 0.003 | 0.18366666666666664 |
| Post | 0.009666666666666667 | 0.16366666666666665 | 0.0 | 0.0003333333333333333 | 0.032 | 0.05566666666666667 | 0.0 | 0.438 | 0.043666666666666666 | 0.004333333333333333 | 0.0 | 0.004333333333333333 | 0.0 | 0.0 | 0.0 | 0.0 | 0.0 | 0.0 | 0.0006666666666666666 | 0.022 | 0.005 | 0.0 | 0.0 | 0.0 | 0.004 | 0.21666666666666667 |
| | None | None | None | None | None | None | None | None | None | None | None | None | None | None | None | None | None | None | None | None | None | None | None | None | None | None |
| Pre | 0.368 | 0.015666666666666666 | 0.032 | 0.085 | 0.09566666666666666 | 0.0003333333333333333 | 0.0 | 0.0023333333333333335 | 0.005666666666666667 | 0.14266666666666666 | 0.0003333333333333333 | 0.021333333333333333 | 0.0 | 0.0 | 0.0 | 0.0 | 0.0 | 0.0006666666666666666 | 0.049666666666666665 | 0.001 | 0.035333333333333335 | 0.0 | 0.002 | 0.0 | 0.0 | 0.14233333333333353 |
| Pre | 0.146 | 0.25333333333333335 | 0.20233333333333334 | 0.014 | 0.07633333333333334 | 0.002 | 0.0 | 0.006333333333333333 | 0.05733333333333333 | 0.056 | 0.0 | 0.012333333333333333 | 0.0026666666666666666 | 0.0 | 0.0 | 0.009333333333333334 | 0.0 | 0.0023333333333333335 | 0.005333333333333333 | 0.005333333333333333 | 0.0003333333333333333 | 0.0 | 0.0036666666666666666 | 0.0003333333333333333 | 0.0 | 0.14466666666666672 |
| FOS | 0.4126666666666667 | 0.1 | 0.143 | 0.043333333333333335 | 0.051333333333333335 | 0.006666666666666667 | 0.0003333333333333333 | 0.019333333333333334 | 0.021666666666666667 | 0.03833333333333333 | 0.0 | 0.0023333333333333335 | 0.0026666666666666666 | 0.0 | 0.0 | 0.0003333333333333333 | 0.0 | 0.007 | 0.0023333333333333335 | 0.0023333333333333335 | 0.005 | 0.0 | 0.009333333333333334 | 0.015333333333333332 | 0.0 | 0.11666666666666681 |
| FOS | 0.32666666666666666 | 0.112 | 0.16766666666666666 | 0.013 | 0.07633333333333334 | 0.003 | 0.0003333333333333333 | 0.022333333333333334 | 0.031 | 0.07933333333333334 | 0.0 | 0.004333333333333333 | 0.0016666666666666668 | 0.0 | 0.0 | 0.0016666666666666668 | 0.0 | 0.003 | 0.001 | 0.0016666666666666668 | 0.0033333333333333335 | 0.0 | 0.008333333333333333 | 0.0003333333333333333 | 0.0 | 0.14300000000000002 |
| Post | 0.23233333333333334 | 0.03666666666666667 | 0.13633333333333333 | 0.047 | 0.127 | 0.0016666666666666668 | 0.0006666666666666666 | 0.004333333333333333 | 0.017666666666666667 | 0.23966666666666667 | 0.0 | 0.014 | 0.0 | 0.0 | 0.0 | 0.0013333333333333333 | 0.0 | 0.0013333333333333333 | 0.022 | 0.003 | 0.029333333333333333 | 0.0 | 0.001 | 0.0 | 0.0 | 0.08466666666666667 |
| Post | 0.167 | 0.04733333333333333 | 0.153 | 0.019333333333333334 | 0.16733333333333333 | 0.001 | 0.0 | 0.019 | 0.018333333333333333 | 0.196 | 0.0 | 0.017666666666666667 | 0.0 | 0.0 | 0.0 | 0.007 | 0.0 | 0.0033333333333333335 | 0.01633333333333333 | 0.006333333333333333 | 0.0003333333333333333 | 0.0 | 0.0023333333333333335 | 0.001 | 0.0 | 0.15733333333333344 |
| | None | None | None | None | None | None | None | None | None | None | None | None | None | None | None | None | None | None | None | None | None | None | None | None | None | None |
| Pre | 0.029666666666666668 | 0.045 | 0.13766666666666666 | 0.07133333333333333 | 0.06933333333333333 | 0.079 | 0.04133333333333333 | 0.06066666666666667 | 0.07066666666666667 | 0.008666666666666666 | 0.12366666666666666 | 0.004666666666666667 | 0.020666666666666667 | 0.033 | 0.0016666666666666668 | 0.001 | 0.010666666666666666 | 0.009666666666666667 | 0.0006666666666666666 | 0.0 | 0.0 | 0.0 | 0.0 | 0.0 | 0.002 | 0.1789999999999996 |
| Pre | 0.027666666666666666 | 0.04733333333333333 | 0.13033333333333333 | 0.10566666666666667 | 0.052 | 0.029 | 0.06333333333333334 | 0.015333333333333332 | 0.042 | 0.004 | 0.15166666666666667 | 0.008666666666666666 | 0.02666666666666667 | 0.07133333333333333 | 0.0 | 0.008666666666666666 | 0.012333333333333333 | 0.003 | 0.0 | 0.0 | 0.0 | 0.0 | 0.0 | 0.0 | 0.0036666666666666666 | 0.19733333333333314 |
| FOS | 0.058666666666666666 | 0.056 | 0.08866666666666667 | 0.09566666666666666 | 0.04 | 0.04666666666666667 | 0.08933333333333333 | 0.06266666666666666 | 0.052 | 0.004666666666666667 | 0.108 | 0.0036666666666666666 | 0.042 | 0.067 | 0.0006666666666666666 | 0.005333333333333333 | 0.015666666666666666 | 0.003 | 0.0 | 0.0 | 0.0 | 0.0 | 0.0 | 0.0 | 0.004 | 0.1563333333333331 |
| FOS | 0.10833333333333334 | 0.023333333333333334 | 0.12433333333333334 | 0.102 | 0.029 | 0.04566666666666667 | 0.14166666666666666 | 0.021333333333333333 | 0.047 | 0.007666666666666666 | 0.12066666666666667 | 0.019 | 0.023333333333333334 | 0.0033333333333333335 | 0.004666666666666667 | 0.0026666666666666666 | 0.011666666666666667 | 0.009 | 0.0003333333333333333 | 0.0 | 0.0 | 0.0 | 0.0 | 0.0003333333333333333 | 0.0023333333333333335 | 0.1523333333333332 |
| Post | 0.034666666666666665 | 0.04066666666666666 | 0.09433333333333334 | 0.10366666666666667 | 0.05533333333333333 | 0.085 | 0.009 | 0.025666666666666667 | 0.057 | 0.01 | 0.22866666666666666 | 0.018666666666666668 | 0.03866666666666667 | 0.03333333333333333 | 0.0006666666666666666 | 0.004333333333333333 | 0.015 | 0.007333333333333333 | 0.0 | 0.0 | 0.0 | 0.0 | 0.0 | 0.0 | 0.004333333333333333 | 0.1336666666666666 |
| Post | 0.09766666666666667 | 0.046 | 0.10433333333333333 | 0.11266666666666666 | 0.04133333333333333 | 0.048 | 0.009333333333333334 | 0.018333333333333333 | 0.03166666666666667 | 0.008333333333333333 | 0.26166666666666666 | 0.010666666666666666 | 0.04533333333333334 | 0.015666666666666666 | 0.0016666666666666668 | 0.0026666666666666666 | 0.017 | 0.0026666666666666666 | 0.0 | 0.0 | 0.0 | 0.0 | 0.0 | 0.0 | 0.0006666666666666666 | 0.12433333333333318 |
| | None | None | None | None | None | None | None | None | None | None | None | None | None | None | None | None | None | None | None | None | None | None | None | None | None | None |
| Pre | 0.02 | 0.106 | 0.07566666666666666 | 0.13266666666666665 | 0.021666666666666667 | 0.027333333333333334 | 0.332 | 0.030333333333333334 | 0.034333333333333334 | 0.016666666666666666 | 0.0 | 0.006 | 0.02666666666666667 | 0.0 | 0.005666666666666667 | 0.008666666666666666 | 0.007333333333333333 | 0.0013333333333333333 | 0.0 | 0.0006666666666666666 | 0.0 | 0.0 | 0.0 | 0.002 | 0.0 | 0.1449999999999999 |
| Pre | 0.132 | 0.052333333333333336 | 0.098 | 0.132 | 0.021666666666666667 | 0.010666666666666666 | 0.3566666666666667 | 0.004 | 0.02 | 0.018666666666666668 | 0.0 | 0.0033333333333333335 | 0.013 | 0.0 | 0.003 | 0.01633333333333333 | 0.007333333333333333 | 0.0013333333333333333 | 0.0 | 0.0003333333333333333 | 0.0 | 0.0 | 0.0 | 0.0036666666666666666 | 0.0 | 0.10566666666666669 |
| FOS | 0.023666666666666666 | 0.09633333333333334 | 0.09366666666666666 | 0.185 | 0.04033333333333333 | 0.021666666666666667 | 0.14 | 0.026 | 0.04533333333333334 | 0.030333333333333334 | 0.0 | 0.015 | 0.020666666666666667 | 0.0 | 0.004 | 0.02033333333333333 | 0.012 | 0.0003333333333333333 | 0.0 | 0.0 | 0.0 | 0.0 | 0.0 | 0.007333333333333333 | 0.0 | 0.21800000000000008 |
| FOS | 0.177 | 0.057666666666666665 | 0.063 | 0.171 | 0.013666666666666667 | 0.027666666666666666 | 0.2693333333333333 | 0.022 | 0.017 | 0.006 | 0.0 | 0.013 | 0.019333333333333334 | 0.0 | 0.02266666666666667 | 0.008333333333333333 | 0.006666666666666667 | 0.0033333333333333335 | 0.0 | 0.0003333333333333333 | 0.0 | 0.0 | 0.0 | 0.0026666666666666666 | 0.0 | 0.09933333333333338 |
| Post | 0.07033333333333333 | 0.13833333333333334 | 0.16866666666666666 | 0.17466666666666666 | 0.033666666666666664 | 0.024 | 0.024666666666666667 | 0.01 | 0.039 | 0.026 | 0.0 | 0.009 | 0.036333333333333336 | 0.0 | 0.01 | 0.011333333333333334 | 0.006 | 0.002 | 0.0 | 0.0 | 0.0006666666666666666 | 0.0 | 0.0 | 0.001 | 0.0 | 0.21433333333333338 |
| Post | 0.093 | 0.058666666666666666 | 0.15733333333333333 | 0.20966666666666667 | 0.03133333333333333 | 0.018 | 0.09233333333333334 | 0.015 | 0.041666666666666664 | 0.018333333333333333 | 0.0 | 0.004666666666666667 | 0.014333333333333333 | 0.0 | 0.0026666666666666666 | 0.042 | 0.007333333333333333 | 0.007333333333333333 | 0.0 | 0.0 | 0.001 | 0.0 | 0.0 | 0.0 | 0.0 | 0.18533333333333335 |
| | None | None | None | None | None | None | None | None | None | None | None | None | None | None | None | None | None | None | None | None | None | None | None | None | None | None |
| Pre | 0.101 | 0.18866666666666668 | 0.042 | 0.18866666666666668 | 0.111 | 0.09366666666666666 | 0.0026666666666666666 | 0.071 | 0.042333333333333334 | 0.04566666666666667 | 0.0 | 0.004666666666666667 | 0.005333333333333333 | 0.0 | 0.0 | 0.004666666666666667 | 0.010333333333333333 | 0.011 | 0.014666666666666666 | 0.011 | 0.0 | 0.0 | 0.0 | 0.0 | 0.0 | 0.05166666666666664 |
| Pre | 0.29033333333333333 | 0.11666666666666667 | 0.16966666666666666 | 0.07966666666666666 | 0.09533333333333334 | 0.066 | 0.0 | 0.058 | 0.03266666666666666 | 0.023666666666666666 | 0.0 | 0.010333333333333333 | 0.003 | 0.0 | 0.0 | 0.006333333333333333 | 0.007 | 0.006666666666666667 | 0.0033333333333333335 | 0.002 | 0.0 | 0.0 | 0.0 | 0.0013333333333333333 | 0.0 | 0.028000000000000025 |
| FOS | 0.152 | 0.14866666666666667 | 0.08466666666666667 | 0.16633333333333333 | 0.15666666666666668 | 0.059 | 0.0 | 0.024 | 0.06266666666666666 | 0.051333333333333335 | 0.0 | 0.0036666666666666666 | 0.009666666666666667 | 0.0 | 0.0 | 0.0023333333333333335 | 0.008666666666666666 | 0.007 | 0.005 | 0.008666666666666666 | 0.0 | 0.0 | 0.0 | 0.0 | 0.0 | 0.04966666666666664 |
| FOS | 0.4116666666666667 | 0.06633333333333333 | 0.118 | 0.11366666666666667 | 0.092 | 0.069 | 0.0003333333333333333 | 0.012666666666666666 | 0.015 | 0.06166666666666667 | 0.0 | 0.0026666666666666666 | 0.0036666666666666666 | 0.0 | 0.0 | 0.0 | 0.0036666666666666666 | 0.004 | 0.003 | 0.0006666666666666666 | 0.0 | 0.0 | 0.0 | 0.0 | 0.0 | 0.021999999999999797 |
| Post | 0.07 | 0.19233333333333333 | 0.11533333333333333 | 0.18433333333333332 | 0.10733333333333334 | 0.06233333333333333 | 0.0006666666666666666 | 0.009333333333333334 | 0.06966666666666667 | 0.051333333333333335 | 0.0 | 0.006 | 0.0023333333333333335 | 0.0 | 0.0 | 0.003 | 0.005 | 0.005 | 0.011333333333333334 | 0.005333333333333333 | 0.0003333333333333333 | 0.0 | 0.0 | 0.0016666666666666668 | 0.0 | 0.09733333333333338 |
| Post | 0.11033333333333334 | 0.13333333333333333 | 0.17166666666666666 | 0.22266666666666668 | 0.122 | 0.06733333333333333 | 0.0 | 0.025333333333333333 | 0.029 | 0.037 | 0.0 | 0.0023333333333333335 | 0.009333333333333334 | 0.0 | 0.0 | 0.0036666666666666666 | 0.007666666666666666 | 0.011666666666666667 | 0.0023333333333333335 | 0.006 | 0.0 | 0.0 | 0.0 | 0.0006666666666666666 | 0.0 | 0.037666666666666515 |
| | None | None | None | None | None | None | None | None | None | None | None | None | None | None | None | None | None | None | None | None | None | None | None | None | None | None |
| Pre | 0.2976666666666667 | 0.08033333333333334 | 0.042 | 0.04133333333333333 | 0.05366666666666667 | 0.20733333333333334 | 0.005 | 0.014 | 0.03933333333333333 | 0.018333333333333333 | 0.0 | 0.004 | 0.02666666666666667 | 0.0 | 0.0 | 0.002 | 0.003 | 0.013 | 0.005 | 0.0 | 0.001 | 0.0 | 0.0 | 0.0003333333333333333 | 0.0 | 0.14600000000000002 |
| Pre | 0.125 | 0.087 | 0.14233333333333334 | 0.082 | 0.11266666666666666 | 0.11233333333333333 | 0.001 | 0.008333333333333333 | 0.037333333333333336 | 0.051333333333333335 | 0.0 | 0.005666666666666667 | 0.030333333333333334 | 0.0003333333333333333 | 0.0 | 0.008666666666666666 | 0.0033333333333333335 | 0.0033333333333333335 | 0.0026666666666666666 | 0.0 | 0.0 | 0.0 | 0.0 | 0.001 | 0.0 | 0.18533333333333335 |
| FOS | 0.494 | 0.030666666666666665 | 0.07433333333333333 | 0.07766666666666666 | 0.048666666666666664 | 0.051333333333333335 | 0.073 | 0.021333333333333333 | 0.011333333333333334 | 0.012333333333333333 | 0.0 | 0.006333333333333333 | 0.017333333333333333 | 0.0 | 0.0 | 0.0 | 0.001 | 0.004666666666666667 | 0.0006666666666666666 | 0.0003333333333333333 | 0.0 | 0.0 | 0.0 | 0.0036666666666666666 | 0.0 | 0.07133333333333347 |
| FOS | 0.43033333333333335 | 0.04933333333333333 | 0.07333333333333333 | 0.06533333333333333 | 0.08233333333333333 | 0.06966666666666667 | 0.009 | 0.01 | 0.021666666666666667 | 0.024 | 0.0 | 0.009666666666666667 | 0.02266666666666667 | 0.0 | 0.0 | 0.0 | 0.004333333333333333 | 0.004666666666666667 | 0.0 | 0.0 | 0.0003333333333333333 | 0.0 | 0.0 | 0.0006666666666666666 | 0.0 | 0.12266666666666648 |
| Post | 0.24266666666666667 | 0.055 | 0.12666666666666668 | 0.08433333333333333 | 0.08433333333333333 | 0.10466666666666667 | 0.014333333333333333 | 0.007666666666666666 | 0.025333333333333333 | 0.03 | 0.0 | 0.025333333333333333 | 0.020666666666666667 | 0.0 | 0.0 | 0.008666666666666666 | 0.0036666666666666666 | 0.006333333333333333 | 0.0 | 0.0 | 0.0003333333333333333 | 0.0 | 0.0 | 0.002 | 0.0 | 0.1579999999999998 |
| Post | 0.2823333333333333 | 0.10633333333333334 | 0.04066666666666666 | 0.06633333333333333 | 0.088 | 0.16633333333333333 | 0.0003333333333333333 | 0.0003333333333333333 | 0.034 | 0.017 | 0.0 | 0.026333333333333334 | 0.042333333333333334 | 0.0 | 0.0 | 0.0016666666666666668 | 0.005 | 0.0 | 0.001 | 0.0 | 0.0003333333333333333 | 0.0 | 0.0 | 0.001 | 0.0 | 0.1206666666666667 |
| | None | None | None | None | None | None | None | None | None | None | None | None | None | None | None | None | None | None | None | None | None | None | None | None | None | None |
| Pre | 0.18133333333333335 | 0.12866666666666668 | 0.105 | 0.006666666666666667 | 0.161 | 0.0 | 0.0 | 0.16633333333333333 | 0.014 | 0.021666666666666667 | 0.0 | 0.017666666666666667 | 0.0 | 0.0 | 0.0 | 0.049 | 0.0 | 0.0 | 0.008666666666666666 | 0.0 | 0.001 | 0.0 | 0.014333333333333333 | 0.0003333333333333333 | 0.0 | 0.1243333333333333 |
| Pre | 0.09866666666666667 | 0.13833333333333334 | 0.11266666666666666 | 0.009666666666666667 | 0.331 | 0.0 | 0.0 | 0.07633333333333334 | 0.028 | 0.057666666666666665 | 0.0 | 0.022333333333333334 | 0.0 | 0.0 | 0.0 | 0.011666666666666667 | 0.0 | 0.0 | 0.005 | 0.0 | 0.0036666666666666666 | 0.0 | 0.017666666666666667 | 0.0003333333333333333 | 0.0 | 0.08699999999999986 |
| FOS | 0.3243333333333333 | 0.073 | 0.06766666666666667 | 0.006666666666666667 | 0.21566666666666667 | 0.0 | 0.0 | 0.111 | 0.013333333333333334 | 0.028666666666666667 | 0.0 | 0.017333333333333333 | 0.0 | 0.0 | 0.0 | 0.012666666666666666 | 0.0 | 0.0 | 0.007 | 0.0 | 0.008 | 0.0 | 0.012666666666666666 | 0.0003333333333333333 | 0.0 | 0.10166666666666668 |
| FOS | 0.45866666666666667 | 0.07633333333333334 | 0.028333333333333332 | 0.006666666666666667 | 0.114 | 0.0 | 0.0 | 0.017 | 0.017333333333333333 | 0.004666666666666667 | 0.0 | 0.019333333333333334 | 0.0 | 0.0 | 0.0 | 0.04733333333333333 | 0.0 | 0.0 | 0.004 | 0.0 | 0.016 | 0.0 | 0.011 | 0.107 | 0.0 | 0.07233333333333325 |
| Post | 0.341 | 0.16266666666666665 | 0.06933333333333333 | 0.014666666666666666 | 0.23866666666666667 | 0.0 | 0.0 | 0.005333333333333333 | 0.025333333333333333 | 0.03766666666666667 | 0.0 | 0.009666666666666667 | 0.0 | 0.0 | 0.0 | 0.01633333333333333 | 0.0 | 0.0 | 0.006333333333333333 | 0.0 | 0.0 | 0.0 | 0.006 | 0.0006666666666666666 | 0.0 | 0.06633333333333324 |
| Post | 0.49133333333333334 | 0.098 | 0.11233333333333333 | 0.044333333333333336 | 0.05466666666666667 | 0.0 | 0.0 | 0.01 | 0.01633333333333333 | 0.01 | 0.0 | 0.03166666666666667 | 0.0 | 0.0 | 0.0 | 0.011666666666666667 | 0.0 | 0.0 | 0.017666666666666667 | 0.0 | 0.006333333333333333 | 0.0 | 0.007 | 0.0006666666666666666 | 0.0 | 0.08799999999999997 |
| | None | None | None | None | None | None | None | None | None | None | None | None | None | None | None | None | None | None | None | None | None | None | None | None | None | None |
| Pre | 0.09266666666666666 | 0.026333333333333334 | 0.06166666666666667 | 0.05533333333333333 | 0.05466666666666667 | 0.0 | 0.37633333333333335 | 0.10066666666666667 | 0.014 | 0.008666666666666666 | 0.0 | 0.006333333333333333 | 0.006 | 0.0 | 0.015333333333333332 | 0.0016666666666666668 | 0.005666666666666667 | 0.0 | 0.001 | 0.0 | 0.0 | 0.04 | 0.020666666666666667 | 0.0 | 0.006666666666666667 | 0.10633333333333306 |
| Pre | 0.25233333333333335 | 0.052 | 0.145 | 0.07366666666666667 | 0.134 | 0.0 | 0.010333333333333333 | 0.011333333333333334 | 0.038 | 0.02 | 0.0 | 0.013333333333333334 | 0.009 | 0.0 | 0.010333333333333333 | 0.0016666666666666668 | 0.006333333333333333 | 0.0 | 0.0013333333333333333 | 0.0 | 0.0 | 0.023666666666666666 | 0.005666666666666667 | 0.0 | 0.0 | 0.19200000000000006 |
| FOS | 0.3456666666666667 | 0.02266666666666667 | 0.07333333333333333 | 0.016666666666666666 | 0.07266666666666667 | 0.0 | 0.058333333333333334 | 0.15933333333333333 | 0.024666666666666667 | 0.015666666666666666 | 0.0 | 0.0023333333333333335 | 0.006333333333333333 | 0.0 | 0.02266666666666667 | 0.0006666666666666666 | 0.0026666666666666666 | 0.0 | 0.0023333333333333335 | 0.0 | 0.0003333333333333333 | 0.021666666666666667 | 0.02 | 0.0 | 0.004666666666666667 | 0.1273333333333332 |
| FOS | 0.464 | 0.034333333333333334 | 0.137 | 0.017666666666666667 | 0.08433333333333333 | 0.0 | 0.009 | 0.022333333333333334 | 0.025333333333333333 | 0.012333333333333333 | 0.0 | 0.005666666666666667 | 0.007666666666666666 | 0.0 | 0.008 | 0.0036666666666666666 | 0.005666666666666667 | 0.0 | 0.002 | 0.0 | 0.0 | 0.043333333333333335 | 0.012333333333333333 | 0.0 | 0.002 | 0.10333333333333317 |
| Post | 0.11066666666666666 | 0.06333333333333334 | 0.19066666666666668 | 0.028 | 0.118 | 0.0 | 0.02 | 0.09866666666666667 | 0.039 | 0.021666666666666667 | 0.0 | 0.0016666666666666668 | 0.010666666666666666 | 0.0 | 0.007333333333333333 | 0.0033333333333333335 | 0.007 | 0.0 | 0.0 | 0.0 | 0.0 | 0.012 | 0.008666666666666666 | 0.0 | 0.0006666666666666666 | 0.2586666666666664 |
| Post | 0.14533333333333334 | 0.07166666666666667 | 0.20666666666666667 | 0.049 | 0.11466666666666667 | 0.0 | 0.014 | 0.06866666666666667 | 0.03966666666666667 | 0.012333333333333333 | 0.0 | 0.0026666666666666666 | 0.013333333333333334 | 0.0 | 0.008333333333333333 | 0.005666666666666667 | 0.01 | 0.0 | 0.0013333333333333333 | 0.0 | 0.0 | 0.027666666666666666 | 0.004 | 0.0 | 0.0013333333333333333 | 0.20366666666666677 |
| | None | None | None | None | None | None | None | None | None | None | None | None | None | None | None | None | None | None | None | None | None | None | None | None | None | None |
| Pre | 0.0 | 0.04566666666666667 | 0.03133333333333333 | 0.06466666666666666 | 0.041666666666666664 | 0.09 | 0.467 | 0.054 | 0.021333333333333333 | 0.0023333333333333335 | 0.021333333333333333 | 0.021 | 0.01633333333333333 | 0.04066666666666666 | 0.0 | 0.0 | 0.003 | 0.004333333333333333 | 0.0003333333333333333 | 0.0033333333333333335 | 0.0 | 0.0 | 0.0 | 0.0 | 0.0 | 0.07166666666666688 |
| Pre | 0.0006666666666666666 | 0.09233333333333334 | 0.07733333333333334 | 0.26866666666666666 | 0.020666666666666667 | 0.10766666666666666 | 0.15933333333333333 | 0.014666666666666666 | 0.049 | 0.006 | 0.03933333333333333 | 0.04133333333333333 | 0.02 | 0.005333333333333333 | 0.0 | 0.0 | 0.002 | 0.0016666666666666668 | 0.0 | 0.0023333333333333335 | 0.0 | 0.0 | 0.0 | 0.0013333333333333333 | 0.0 | 0.09033333333333327 |
| FOS | 0.0006666666666666666 | 0.029333333333333333 | 0.008333333333333333 | 0.015666666666666666 | 0.009666666666666667 | 0.12066666666666667 | 0.489 | 0.0016666666666666668 | 0.013333333333333334 | 0.0013333333333333333 | 0.059666666666666666 | 0.048666666666666664 | 0.013333333333333334 | 0.146 | 0.0 | 0.0 | 0.001 | 0.0006666666666666666 | 0.0003333333333333333 | 0.003 | 0.0016666666666666668 | 0.0 | 0.0 | 0.0 | 0.0 | 0.03600000000000003 |
| FOS | 0.0023333333333333335 | 0.014666666666666666 | 0.0023333333333333335 | 0.009 | 0.022333333333333334 | 0.17366666666666666 | 0.48533333333333334 | 0.0023333333333333335 | 0.004333333333333333 | 0.0003333333333333333 | 0.018333333333333333 | 0.049 | 0.008666666666666666 | 0.15233333333333332 | 0.0 | 0.0 | 0.0003333333333333333 | 0.0 | 0.0 | 0.005333333333333333 | 0.002 | 0.0 | 0.0 | 0.0 | 0.0 | 0.04733333333333345 |
| Post | 0.0023333333333333335 | 0.077 | 0.072 | 0.085 | 0.037 | 0.18666666666666668 | 0.17366666666666666 | 0.028 | 0.03966666666666667 | 0.003 | 0.06966666666666667 | 0.08633333333333333 | 0.02266666666666667 | 0.0013333333333333333 | 0.0 | 0.0 | 0.002 | 0.005666666666666667 | 0.0006666666666666666 | 0.002 | 0.0006666666666666666 | 0.0 | 0.0 | 0.0 | 0.0 | 0.10466666666666657 |
| Post | 0.0 | 0.097 | 0.029 | 0.16 | 0.03966666666666667 | 0.289 | 0.009 | 0.0016666666666666668 | 0.03966666666666667 | 0.005 | 0.11166666666666666 | 0.11033333333333334 | 0.03233333333333333 | 0.0 | 0.0 | 0.0 | 0.0023333333333333335 | 0.0 | 0.0 | 0.0 | 0.0 | 0.0003333333333333333 | 0.0 | 0.0 | 0.0 | 0.07299999999999995 |
| | None | None | None | None | None | None | None | None | None | None | None | None | None | None | None | None | None | None | None | None | None | None | None | None | None | None |
| Pre | 0.04566666666666667 | 0.035666666666666666 | 0.17233333333333334 | 0.04633333333333333 | 0.17166666666666666 | 0.04033333333333333 | 0.0 | 0.19266666666666668 | 0.035333333333333335 | 0.010666666666666666 | 0.0 | 0.004333333333333333 | 0.017 | 0.0 | 0.0 | 0.0016666666666666668 | 0.0013333333333333333 | 0.07733333333333334 | 0.0 | 0.0016666666666666668 | 0.0 | 0.0 | 0.0 | 0.0023333333333333335 | 0.0 | 0.1436666666666666 |
| Pre | 0.023666666666666666 | 0.11466666666666667 | 0.09666666666666666 | 0.112 | 0.167 | 0.09733333333333333 | 0.0 | 0.039 | 0.06333333333333334 | 0.03133333333333333 | 0.0 | 0.021 | 0.025 | 0.0 | 0.0 | 0.0 | 0.0033333333333333335 | 0.011 | 0.001 | 0.002 | 0.0006666666666666666 | 0.0 | 0.0 | 0.0 | 0.0 | 0.19099999999999995 |
| FOS | 0.5653333333333334 | 0.025 | 0.08566666666666667 | 0.011666666666666667 | 0.045 | 0.151 | 0.0 | 0.025 | 0.02033333333333333 | 0.010333333333333333 | 0.0 | 0.0026666666666666666 | 0.013666666666666667 | 0.0 | 0.0 | 0.0003333333333333333 | 0.004333333333333333 | 0.0003333333333333333 | 0.0 | 0.0 | 0.0 | 0.0 | 0.0 | 0.0 | 0.0 | 0.03933333333333322 |
| FOS | 0.4036666666666667 | 0.035333333333333335 | 0.13133333333333333 | 0.015 | 0.123 | 0.10266666666666667 | 0.0 | 0.029333333333333333 | 0.025 | 0.019666666666666666 | 0.0 | 0.0026666666666666666 | 0.014333333333333333 | 0.0 | 0.0 | 0.0003333333333333333 | 0.0023333333333333335 | 0.0006666666666666666 | 0.0006666666666666666 | 0.0 | 0.0 | 0.0 | 0.0 | 0.0 | 0.0 | 0.09399999999999997 |
| Post | 0.124 | 0.08933333333333333 | 0.15066666666666667 | 0.07633333333333334 | 0.15433333333333332 | 0.09366666666666666 | 0.0 | 0.009 | 0.058 | 0.03 | 0.0 | 0.004333333333333333 | 0.022333333333333334 | 0.0 | 0.0 | 0.001 | 0.001 | 0.0016666666666666668 | 0.001 | 0.0013333333333333333 | 0.0016666666666666668 | 0.0 | 0.0 | 0.0006666666666666666 | 0.0 | 0.17966666666666653 |
| Post | 0.059666666666666666 | 0.09133333333333334 | 0.15233333333333332 | 0.08866666666666667 | 0.207 | 0.07933333333333334 | 0.0 | 0.009333333333333334 | 0.05433333333333333 | 0.02266666666666667 | 0.0 | 0.002 | 0.024333333333333332 | 0.0 | 0.0 | 0.001 | 0.001 | 0.0036666666666666666 | 0.0003333333333333333 | 0.0006666666666666666 | 0.0 | 0.0 | 0.0 | 0.0003333333333333333 | 0.0 | 0.20200000000000007 |
| | None | None | None | None | None | None | None | None | None | None | None | None | None | None | None | None | None | None | None | None | None | None | None | None | None | None |
| Pre | 0.121 | 0.112 | 0.0013333333333333333 | 0.2713333333333333 | 0.07533333333333334 | 0.08233333333333333 | 0.0006666666666666666 | 0.01 | 0.041 | 0.019 | 0.0 | 0.02033333333333333 | 0.016 | 0.0 | 0.010666666666666666 | 0.0 | 0.009333333333333334 | 0.0016666666666666668 | 0.001 | 0.0 | 0.021 | 0.0 | 0.0 | 0.0 | 0.0 | 0.18599999999999983 |
| Pre | 0.221 | 0.11233333333333333 | 0.0026666666666666666 | 0.083 | 0.03933333333333333 | 0.071 | 0.030333333333333334 | 0.17366666666666666 | 0.015 | 0.014333333333333333 | 0.0 | 0.062 | 0.005333333333333333 | 0.0 | 0.07866666666666666 | 0.0006666666666666666 | 0.0023333333333333335 | 0.015666666666666666 | 0.0013333333333333333 | 0.0 | 0.0036666666666666666 | 0.0 | 0.0 | 0.0 | 0.0016666666666666668 | 0.06599999999999995 |
| FOS | 0.4846666666666667 | 0.044 | 0.013333333333333334 | 0.10166666666666667 | 0.028 | 0.08133333333333333 | 0.011333333333333334 | 0.008 | 0.014333333333333333 | 0.0033333333333333335 | 0.0 | 0.005333333333333333 | 0.006666666666666667 | 0.0 | 0.14666666666666667 | 0.0 | 0.008333333333333333 | 0.001 | 0.0 | 0.0013333333333333333 | 0.0016666666666666668 | 0.0 | 0.0 | 0.0 | 0.011666666666666667 | 0.02733333333333321 |
| FOS | 0.192 | 0.07533333333333334 | 0.037333333333333336 | 0.146 | 0.04733333333333333 | 0.098 | 0.093 | 0.08166666666666667 | 0.024333333333333332 | 0.012 | 0.0 | 0.014 | 0.010666666666666666 | 0.0 | 0.06966666666666667 | 0.0 | 0.007333333333333333 | 0.01 | 0.0023333333333333335 | 0.001 | 0.0003333333333333333 | 0.0 | 0.0 | 0.0 | 0.0026666666666666666 | 0.07500000000000007 |
| Post | 0.25166666666666665 | 0.12233333333333334 | 0.005 | 0.13533333333333333 | 0.029 | 0.082 | 0.0003333333333333333 | 0.0033333333333333335 | 0.011666666666666667 | 0.009666666666666667 | 0.0 | 0.023666666666666666 | 0.010333333333333333 | 0.0 | 0.0033333333333333335 | 0.0 | 0.006333333333333333 | 0.001 | 0.0003333333333333333 | 0.0 | 0.0003333333333333333 | 0.0 | 0.0 | 0.0 | 0.0023333333333333335 | 0.30200000000000027 |
| Post | 0.09433333333333334 | 0.11 | 0.035666666666666666 | 0.3413333333333333 | 0.06566666666666666 | 0.09466666666666666 | 0.0016666666666666668 | 0.035666666666666666 | 0.035333333333333335 | 0.021 | 0.0 | 0.006666666666666667 | 0.011333333333333334 | 0.0 | 0.0023333333333333335 | 0.0003333333333333333 | 0.010333333333333333 | 0.003 | 0.005 | 0.0006666666666666666 | 0.0003333333333333333 | 0.0 | 0.0 | 0.0 | 0.0016666666666666668 | 0.12300000000000011 |
Relative abundance
ID01
ID02
ID03
ID04
ID05
ID06
ID07
ID08
ID09
ID10
ID11
### Chart
| Category | Bifidobacterium | Ruminococcus | Faecalibacterium | Eubacterium | Clostridium | Collinsella | Prevotella | Bacteroides | Blautia | Anaerostipes | Catenibacterium | Streptococcus | Dorea | Megamonas | Mitsuokella | Roseburia | Coprococcus | Parabacteroides | Eggerthella | Phascolarctobacterium | Escherichia | Selenomonas | Dialister | Lactobacillus | Acidaminococcus | LowAbundance |
|---|---|---|---|---|---|---|---|---|---|---|---|---|---|---|---|---|---|---|---|---|---|---|---|---|---|---|
| Pre | 0.04066666666666666 | 0.16966666666666666 | 0.0 | 0.0 | 0.04833333333333333 | 0.06733333333333333 | 0.0 | 0.432 | 0.059333333333333335 | 0.001 | 0.0 | 0.0 | 0.0 | 0.0 | 0.0 | 0.0 | 0.0 | 0.0 | 0.0026666666666666666 | 0.035 | 0.0023333333333333335 | 0.0 | 0.0 | 0.0003333333333333333 | 0.018 | 0.1233333333333333 |
| Pre | 0.079 | 0.148 | 0.0 | 0.0003333333333333333 | 0.049 | 0.32666666666666666 | 0.0006666666666666666 | 0.0023333333333333335 | 0.158 | 0.004 | 0.0 | 0.005666666666666667 | 0.0 | 0.0 | 0.0 | 0.0 | 0.0 | 0.0 | 0.013 | 0.013333333333333334 | 0.003 | 0.0 | 0.0 | 0.0006666666666666666 | 0.0 | 0.19633333333333325 |
| FOS | 0.174 | 0.328 | 0.0 | 0.0 | 0.006666666666666667 | 0.050333333333333334 | 0.0006666666666666666 | 0.263 | 0.04133333333333333 | 0.0006666666666666666 | 0.0 | 0.0 | 0.0 | 0.0 | 0.0 | 0.0 | 0.0 | 0.0 | 0.001 | 0.020666666666666667 | 0.004333333333333333 | 0.0 | 0.0 | 0.0 | 0.007666666666666666 | 0.10166666666666657 |
| FOS | 0.203 | 0.32366666666666666 | 0.0003333333333333333 | 0.0 | 0.028 | 0.15366666666666667 | 0.0003333333333333333 | 0.035333333333333335 | 0.039 | 0.0 | 0.0 | 0.005 | 0.0 | 0.0 | 0.0 | 0.0 | 0.0 | 0.0 | 0.0006666666666666666 | 0.032 | 0.030666666666666665 | 0.0 | 0.0 | 0.0 | 0.024666666666666667 | 0.12366666666666681 |
| Post | 0.07766666666666666 | 0.33166666666666667 | 0.0 | 0.0 | 0.035 | 0.12133333333333333 | 0.0003333333333333333 | 0.135 | 0.07566666666666666 | 0.0016666666666666668 | 0.0 | 0.0006666666666666666 | 0.0 | 0.0 | 0.0 | 0.0 | 0.0 | 0.0 | 0.014333333333333333 | 0.017333333333333333 | 0.0026666666666666666 | 0.0 | 0.0 | 0.0 | 0.003 | 0.18366666666666664 |
| Post | 0.009666666666666667 | 0.16366666666666665 | 0.0 | 0.0003333333333333333 | 0.032 | 0.05566666666666667 | 0.0 | 0.438 | 0.043666666666666666 | 0.004333333333333333 | 0.0 | 0.004333333333333333 | 0.0 | 0.0 | 0.0 | 0.0 | 0.0 | 0.0 | 0.0006666666666666666 | 0.022 | 0.005 | 0.0 | 0.0 | 0.0 | 0.004 | 0.21666666666666667 |
| | None | None | None | None | None | None | None | None | None | None | None | None | None | None | None | None | None | None | None | None | None | None | None | None | None | None |
| Pre | 0.368 | 0.015666666666666666 | 0.032 | 0.085 | 0.09566666666666666 | 0.0003333333333333333 | 0.0 | 0.0023333333333333335 | 0.005666666666666667 | 0.14266666666666666 | 0.0003333333333333333 | 0.021333333333333333 | 0.0 | 0.0 | 0.0 | 0.0 | 0.0 | 0.0006666666666666666 | 0.049666666666666665 | 0.001 | 0.035333333333333335 | 0.0 | 0.002 | 0.0 | 0.0 | 0.14233333333333353 |
| Pre | 0.146 | 0.25333333333333335 | 0.20233333333333334 | 0.014 | 0.07633333333333334 | 0.002 | 0.0 | 0.006333333333333333 | 0.05733333333333333 | 0.056 | 0.0 | 0.012333333333333333 | 0.0026666666666666666 | 0.0 | 0.0 | 0.009333333333333334 | 0.0 | 0.0023333333333333335 | 0.005333333333333333 | 0.005333333333333333 | 0.0003333333333333333 | 0.0 | 0.0036666666666666666 | 0.0003333333333333333 | 0.0 | 0.14466666666666672 |
| FOS | 0.4126666666666667 | 0.1 | 0.143 | 0.043333333333333335 | 0.051333333333333335 | 0.006666666666666667 | 0.0003333333333333333 | 0.019333333333333334 | 0.021666666666666667 | 0.03833333333333333 | 0.0 | 0.0023333333333333335 | 0.0026666666666666666 | 0.0 | 0.0 | 0.0003333333333333333 | 0.0 | 0.007 | 0.0023333333333333335 | 0.0023333333333333335 | 0.005 | 0.0 | 0.009333333333333334 | 0.015333333333333332 | 0.0 | 0.11666666666666681 |
| FOS | 0.32666666666666666 | 0.112 | 0.16766666666666666 | 0.013 | 0.07633333333333334 | 0.003 | 0.0003333333333333333 | 0.022333333333333334 | 0.031 | 0.07933333333333334 | 0.0 | 0.004333333333333333 | 0.0016666666666666668 | 0.0 | 0.0 | 0.0016666666666666668 | 0.0 | 0.003 | 0.001 | 0.0016666666666666668 | 0.0033333333333333335 | 0.0 | 0.008333333333333333 | 0.0003333333333333333 | 0.0 | 0.14300000000000002 |
| Post | 0.23233333333333334 | 0.03666666666666667 | 0.13633333333333333 | 0.047 | 0.127 | 0.0016666666666666668 | 0.0006666666666666666 | 0.004333333333333333 | 0.017666666666666667 | 0.23966666666666667 | 0.0 | 0.014 | 0.0 | 0.0 | 0.0 | 0.0013333333333333333 | 0.0 | 0.0013333333333333333 | 0.022 | 0.003 | 0.029333333333333333 | 0.0 | 0.001 | 0.0 | 0.0 | 0.08466666666666667 |
| Post | 0.167 | 0.04733333333333333 | 0.153 | 0.019333333333333334 | 0.16733333333333333 | 0.001 | 0.0 | 0.019 | 0.018333333333333333 | 0.196 | 0.0 | 0.017666666666666667 | 0.0 | 0.0 | 0.0 | 0.007 | 0.0 | 0.0033333333333333335 | 0.01633333333333333 | 0.006333333333333333 | 0.0003333333333333333 | 0.0 | 0.0023333333333333335 | 0.001 | 0.0 | 0.15733333333333344 |
| | None | None | None | None | None | None | None | None | None | None | None | None | None | None | None | None | None | None | None | None | None | None | None | None | None | None |
| Pre | 0.029666666666666668 | 0.045 | 0.13766666666666666 | 0.07133333333333333 | 0.06933333333333333 | 0.079 | 0.04133333333333333 | 0.06066666666666667 | 0.07066666666666667 | 0.008666666666666666 | 0.12366666666666666 | 0.004666666666666667 | 0.020666666666666667 | 0.033 | 0.0016666666666666668 | 0.001 | 0.010666666666666666 | 0.009666666666666667 | 0.0006666666666666666 | 0.0 | 0.0 | 0.0 | 0.0 | 0.0 | 0.002 | 0.1789999999999996 |
| Pre | 0.027666666666666666 | 0.04733333333333333 | 0.13033333333333333 | 0.10566666666666667 | 0.052 | 0.029 | 0.06333333333333334 | 0.015333333333333332 | 0.042 | 0.004 | 0.15166666666666667 | 0.008666666666666666 | 0.02666666666666667 | 0.07133333333333333 | 0.0 | 0.008666666666666666 | 0.012333333333333333 | 0.003 | 0.0 | 0.0 | 0.0 | 0.0 | 0.0 | 0.0 | 0.0036666666666666666 | 0.19733333333333314 |
| FOS | 0.058666666666666666 | 0.056 | 0.08866666666666667 | 0.09566666666666666 | 0.04 | 0.04666666666666667 | 0.08933333333333333 | 0.06266666666666666 | 0.052 | 0.004666666666666667 | 0.108 | 0.0036666666666666666 | 0.042 | 0.067 | 0.0006666666666666666 | 0.005333333333333333 | 0.015666666666666666 | 0.003 | 0.0 | 0.0 | 0.0 | 0.0 | 0.0 | 0.0 | 0.004 | 0.1563333333333331 |
| FOS | 0.10833333333333334 | 0.023333333333333334 | 0.12433333333333334 | 0.102 | 0.029 | 0.04566666666666667 | 0.14166666666666666 | 0.021333333333333333 | 0.047 | 0.007666666666666666 | 0.12066666666666667 | 0.019 | 0.023333333333333334 | 0.0033333333333333335 | 0.004666666666666667 | 0.0026666666666666666 | 0.011666666666666667 | 0.009 | 0.0003333333333333333 | 0.0 | 0.0 | 0.0 | 0.0 | 0.0003333333333333333 | 0.0023333333333333335 | 0.1523333333333332 |
| Post | 0.034666666666666665 | 0.04066666666666666 | 0.09433333333333334 | 0.10366666666666667 | 0.05533333333333333 | 0.085 | 0.009 | 0.025666666666666667 | 0.057 | 0.01 | 0.22866666666666666 | 0.018666666666666668 | 0.03866666666666667 | 0.03333333333333333 | 0.0006666666666666666 | 0.004333333333333333 | 0.015 | 0.007333333333333333 | 0.0 | 0.0 | 0.0 | 0.0 | 0.0 | 0.0 | 0.004333333333333333 | 0.1336666666666666 |
| Post | 0.09766666666666667 | 0.046 | 0.10433333333333333 | 0.11266666666666666 | 0.04133333333333333 | 0.048 | 0.009333333333333334 | 0.018333333333333333 | 0.03166666666666667 | 0.008333333333333333 | 0.26166666666666666 | 0.010666666666666666 | 0.04533333333333334 | 0.015666666666666666 | 0.0016666666666666668 | 0.0026666666666666666 | 0.017 | 0.0026666666666666666 | 0.0 | 0.0 | 0.0 | 0.0 | 0.0 | 0.0 | 0.0006666666666666666 | 0.12433333333333318 |
| | None | None | None | None | None | None | None | None | None | None | None | None | None | None | None | None | None | None | None | None | None | None | None | None | None | None |
| Pre | 0.02 | 0.106 | 0.07566666666666666 | 0.13266666666666665 | 0.021666666666666667 | 0.027333333333333334 | 0.332 | 0.030333333333333334 | 0.034333333333333334 | 0.016666666666666666 | 0.0 | 0.006 | 0.02666666666666667 | 0.0 | 0.005666666666666667 | 0.008666666666666666 | 0.007333333333333333 | 0.0013333333333333333 | 0.0 | 0.0006666666666666666 | 0.0 | 0.0 | 0.0 | 0.002 | 0.0 | 0.1449999999999999 |
| Pre | 0.132 | 0.052333333333333336 | 0.098 | 0.132 | 0.021666666666666667 | 0.010666666666666666 | 0.3566666666666667 | 0.004 | 0.02 | 0.018666666666666668 | 0.0 | 0.0033333333333333335 | 0.013 | 0.0 | 0.003 | 0.01633333333333333 | 0.007333333333333333 | 0.0013333333333333333 | 0.0 | 0.0003333333333333333 | 0.0 | 0.0 | 0.0 | 0.0036666666666666666 | 0.0 | 0.10566666666666669 |
| FOS | 0.023666666666666666 | 0.09633333333333334 | 0.09366666666666666 | 0.185 | 0.04033333333333333 | 0.021666666666666667 | 0.14 | 0.026 | 0.04533333333333334 | 0.030333333333333334 | 0.0 | 0.015 | 0.020666666666666667 | 0.0 | 0.004 | 0.02033333333333333 | 0.012 | 0.0003333333333333333 | 0.0 | 0.0 | 0.0 | 0.0 | 0.0 | 0.007333333333333333 | 0.0 | 0.21800000000000008 |
| FOS | 0.177 | 0.057666666666666665 | 0.063 | 0.171 | 0.013666666666666667 | 0.027666666666666666 | 0.2693333333333333 | 0.022 | 0.017 | 0.006 | 0.0 | 0.013 | 0.019333333333333334 | 0.0 | 0.02266666666666667 | 0.008333333333333333 | 0.006666666666666667 | 0.0033333333333333335 | 0.0 | 0.0003333333333333333 | 0.0 | 0.0 | 0.0 | 0.0026666666666666666 | 0.0 | 0.09933333333333338 |
| Post | 0.07033333333333333 | 0.13833333333333334 | 0.16866666666666666 | 0.17466666666666666 | 0.033666666666666664 | 0.024 | 0.024666666666666667 | 0.01 | 0.039 | 0.026 | 0.0 | 0.009 | 0.036333333333333336 | 0.0 | 0.01 | 0.011333333333333334 | 0.006 | 0.002 | 0.0 | 0.0 | 0.0006666666666666666 | 0.0 | 0.0 | 0.001 | 0.0 | 0.21433333333333338 |
| Post | 0.093 | 0.058666666666666666 | 0.15733333333333333 | 0.20966666666666667 | 0.03133333333333333 | 0.018 | 0.09233333333333334 | 0.015 | 0.041666666666666664 | 0.018333333333333333 | 0.0 | 0.004666666666666667 | 0.014333333333333333 | 0.0 | 0.0026666666666666666 | 0.042 | 0.007333333333333333 | 0.007333333333333333 | 0.0 | 0.0 | 0.001 | 0.0 | 0.0 | 0.0 | 0.0 | 0.18533333333333335 |
| | None | None | None | None | None | None | None | None | None | None | None | None | None | None | None | None | None | None | None | None | None | None | None | None | None | None |
| Pre | 0.101 | 0.18866666666666668 | 0.042 | 0.18866666666666668 | 0.111 | 0.09366666666666666 | 0.0026666666666666666 | 0.071 | 0.042333333333333334 | 0.04566666666666667 | 0.0 | 0.004666666666666667 | 0.005333333333333333 | 0.0 | 0.0 | 0.004666666666666667 | 0.010333333333333333 | 0.011 | 0.014666666666666666 | 0.011 | 0.0 | 0.0 | 0.0 | 0.0 | 0.0 | 0.05166666666666664 |
| Pre | 0.29033333333333333 | 0.11666666666666667 | 0.16966666666666666 | 0.07966666666666666 | 0.09533333333333334 | 0.066 | 0.0 | 0.058 | 0.03266666666666666 | 0.023666666666666666 | 0.0 | 0.010333333333333333 | 0.003 | 0.0 | 0.0 | 0.006333333333333333 | 0.007 | 0.006666666666666667 | 0.0033333333333333335 | 0.002 | 0.0 | 0.0 | 0.0 | 0.0013333333333333333 | 0.0 | 0.028000000000000025 |
| FOS | 0.152 | 0.14866666666666667 | 0.08466666666666667 | 0.16633333333333333 | 0.15666666666666668 | 0.059 | 0.0 | 0.024 | 0.06266666666666666 | 0.051333333333333335 | 0.0 | 0.0036666666666666666 | 0.009666666666666667 | 0.0 | 0.0 | 0.0023333333333333335 | 0.008666666666666666 | 0.007 | 0.005 | 0.008666666666666666 | 0.0 | 0.0 | 0.0 | 0.0 | 0.0 | 0.04966666666666664 |
| FOS | 0.4116666666666667 | 0.06633333333333333 | 0.118 | 0.11366666666666667 | 0.092 | 0.069 | 0.0003333333333333333 | 0.012666666666666666 | 0.015 | 0.06166666666666667 | 0.0 | 0.0026666666666666666 | 0.0036666666666666666 | 0.0 | 0.0 | 0.0 | 0.0036666666666666666 | 0.004 | 0.003 | 0.0006666666666666666 | 0.0 | 0.0 | 0.0 | 0.0 | 0.0 | 0.021999999999999797 |
| Post | 0.07 | 0.19233333333333333 | 0.11533333333333333 | 0.18433333333333332 | 0.10733333333333334 | 0.06233333333333333 | 0.0006666666666666666 | 0.009333333333333334 | 0.06966666666666667 | 0.051333333333333335 | 0.0 | 0.006 | 0.0023333333333333335 | 0.0 | 0.0 | 0.003 | 0.005 | 0.005 | 0.011333333333333334 | 0.005333333333333333 | 0.0003333333333333333 | 0.0 | 0.0 | 0.0016666666666666668 | 0.0 | 0.09733333333333338 |
| Post | 0.11033333333333334 | 0.13333333333333333 | 0.17166666666666666 | 0.22266666666666668 | 0.122 | 0.06733333333333333 | 0.0 | 0.025333333333333333 | 0.029 | 0.037 | 0.0 | 0.0023333333333333335 | 0.009333333333333334 | 0.0 | 0.0 | 0.0036666666666666666 | 0.007666666666666666 | 0.011666666666666667 | 0.0023333333333333335 | 0.006 | 0.0 | 0.0 | 0.0 | 0.0006666666666666666 | 0.0 | 0.037666666666666515 |
| | None | None | None | None | None | None | None | None | None | None | None | None | None | None | None | None | None | None | None | None | None | None | None | None | None | None |
| Pre | 0.2976666666666667 | 0.08033333333333334 | 0.042 | 0.04133333333333333 | 0.05366666666666667 | 0.20733333333333334 | 0.005 | 0.014 | 0.03933333333333333 | 0.018333333333333333 | 0.0 | 0.004 | 0.02666666666666667 | 0.0 | 0.0 | 0.002 | 0.003 | 0.013 | 0.005 | 0.0 | 0.001 | 0.0 | 0.0 | 0.0003333333333333333 | 0.0 | 0.14600000000000002 |
| Pre | 0.125 | 0.087 | 0.14233333333333334 | 0.082 | 0.11266666666666666 | 0.11233333333333333 | 0.001 | 0.008333333333333333 | 0.037333333333333336 | 0.051333333333333335 | 0.0 | 0.005666666666666667 | 0.030333333333333334 | 0.0003333333333333333 | 0.0 | 0.008666666666666666 | 0.0033333333333333335 | 0.0033333333333333335 | 0.0026666666666666666 | 0.0 | 0.0 | 0.0 | 0.0 | 0.001 | 0.0 | 0.18533333333333335 |
| FOS | 0.494 | 0.030666666666666665 | 0.07433333333333333 | 0.07766666666666666 | 0.048666666666666664 | 0.051333333333333335 | 0.073 | 0.021333333333333333 | 0.011333333333333334 | 0.012333333333333333 | 0.0 | 0.006333333333333333 | 0.017333333333333333 | 0.0 | 0.0 | 0.0 | 0.001 | 0.004666666666666667 | 0.0006666666666666666 | 0.0003333333333333333 | 0.0 | 0.0 | 0.0 | 0.0036666666666666666 | 0.0 | 0.07133333333333347 |
| FOS | 0.43033333333333335 | 0.04933333333333333 | 0.07333333333333333 | 0.06533333333333333 | 0.08233333333333333 | 0.06966666666666667 | 0.009 | 0.01 | 0.021666666666666667 | 0.024 | 0.0 | 0.009666666666666667 | 0.02266666666666667 | 0.0 | 0.0 | 0.0 | 0.004333333333333333 | 0.004666666666666667 | 0.0 | 0.0 | 0.0003333333333333333 | 0.0 | 0.0 | 0.0006666666666666666 | 0.0 | 0.12266666666666648 |
| Post | 0.24266666666666667 | 0.055 | 0.12666666666666668 | 0.08433333333333333 | 0.08433333333333333 | 0.10466666666666667 | 0.014333333333333333 | 0.007666666666666666 | 0.025333333333333333 | 0.03 | 0.0 | 0.025333333333333333 | 0.020666666666666667 | 0.0 | 0.0 | 0.008666666666666666 | 0.0036666666666666666 | 0.006333333333333333 | 0.0 | 0.0 | 0.0003333333333333333 | 0.0 | 0.0 | 0.002 | 0.0 | 0.1579999999999998 |
| Post | 0.2823333333333333 | 0.10633333333333334 | 0.04066666666666666 | 0.06633333333333333 | 0.088 | 0.16633333333333333 | 0.0003333333333333333 | 0.0003333333333333333 | 0.034 | 0.017 | 0.0 | 0.026333333333333334 | 0.042333333333333334 | 0.0 | 0.0 | 0.0016666666666666668 | 0.005 | 0.0 | 0.001 | 0.0 | 0.0003333333333333333 | 0.0 | 0.0 | 0.001 | 0.0 | 0.1206666666666667 |
| | None | None | None | None | None | None | None | None | None | None | None | None | None | None | None | None | None | None | None | None | None | None | None | None | None | None |
| Pre | 0.18133333333333335 | 0.12866666666666668 | 0.105 | 0.006666666666666667 | 0.161 | 0.0 | 0.0 | 0.16633333333333333 | 0.014 | 0.021666666666666667 | 0.0 | 0.017666666666666667 | 0.0 | 0.0 | 0.0 | 0.049 | 0.0 | 0.0 | 0.008666666666666666 | 0.0 | 0.001 | 0.0 | 0.014333333333333333 | 0.0003333333333333333 | 0.0 | 0.1243333333333333 |
| Pre | 0.09866666666666667 | 0.13833333333333334 | 0.11266666666666666 | 0.009666666666666667 | 0.331 | 0.0 | 0.0 | 0.07633333333333334 | 0.028 | 0.057666666666666665 | 0.0 | 0.022333333333333334 | 0.0 | 0.0 | 0.0 | 0.011666666666666667 | 0.0 | 0.0 | 0.005 | 0.0 | 0.0036666666666666666 | 0.0 | 0.017666666666666667 | 0.0003333333333333333 | 0.0 | 0.08699999999999986 |
| FOS | 0.3243333333333333 | 0.073 | 0.06766666666666667 | 0.006666666666666667 | 0.21566666666666667 | 0.0 | 0.0 | 0.111 | 0.013333333333333334 | 0.028666666666666667 | 0.0 | 0.017333333333333333 | 0.0 | 0.0 | 0.0 | 0.012666666666666666 | 0.0 | 0.0 | 0.007 | 0.0 | 0.008 | 0.0 | 0.012666666666666666 | 0.0003333333333333333 | 0.0 | 0.10166666666666668 |
| FOS | 0.45866666666666667 | 0.07633333333333334 | 0.028333333333333332 | 0.006666666666666667 | 0.114 | 0.0 | 0.0 | 0.017 | 0.017333333333333333 | 0.004666666666666667 | 0.0 | 0.019333333333333334 | 0.0 | 0.0 | 0.0 | 0.04733333333333333 | 0.0 | 0.0 | 0.004 | 0.0 | 0.016 | 0.0 | 0.011 | 0.107 | 0.0 | 0.07233333333333325 |
| Post | 0.341 | 0.16266666666666665 | 0.06933333333333333 | 0.014666666666666666 | 0.23866666666666667 | 0.0 | 0.0 | 0.005333333333333333 | 0.025333333333333333 | 0.03766666666666667 | 0.0 | 0.009666666666666667 | 0.0 | 0.0 | 0.0 | 0.01633333333333333 | 0.0 | 0.0 | 0.006333333333333333 | 0.0 | 0.0 | 0.0 | 0.006 | 0.0006666666666666666 | 0.0 | 0.06633333333333324 |
| Post | 0.49133333333333334 | 0.098 | 0.11233333333333333 | 0.044333333333333336 | 0.05466666666666667 | 0.0 | 0.0 | 0.01 | 0.01633333333333333 | 0.01 | 0.0 | 0.03166666666666667 | 0.0 | 0.0 | 0.0 | 0.011666666666666667 | 0.0 | 0.0 | 0.017666666666666667 | 0.0 | 0.006333333333333333 | 0.0 | 0.007 | 0.0006666666666666666 | 0.0 | 0.08799999999999997 |
| | None | None | None | None | None | None | None | None | None | None | None | None | None | None | None | None | None | None | None | None | None | None | None | None | None | None |
| Pre | 0.09266666666666666 | 0.026333333333333334 | 0.06166666666666667 | 0.05533333333333333 | 0.05466666666666667 | 0.0 | 0.37633333333333335 | 0.10066666666666667 | 0.014 | 0.008666666666666666 | 0.0 | 0.006333333333333333 | 0.006 | 0.0 | 0.015333333333333332 | 0.0016666666666666668 | 0.005666666666666667 | 0.0 | 0.001 | 0.0 | 0.0 | 0.04 | 0.020666666666666667 | 0.0 | 0.006666666666666667 | 0.10633333333333306 |
| Pre | 0.25233333333333335 | 0.052 | 0.145 | 0.07366666666666667 | 0.134 | 0.0 | 0.010333333333333333 | 0.011333333333333334 | 0.038 | 0.02 | 0.0 | 0.013333333333333334 | 0.009 | 0.0 | 0.010333333333333333 | 0.0016666666666666668 | 0.006333333333333333 | 0.0 | 0.0013333333333333333 | 0.0 | 0.0 | 0.023666666666666666 | 0.005666666666666667 | 0.0 | 0.0 | 0.19200000000000006 |
| FOS | 0.3456666666666667 | 0.02266666666666667 | 0.07333333333333333 | 0.016666666666666666 | 0.07266666666666667 | 0.0 | 0.058333333333333334 | 0.15933333333333333 | 0.024666666666666667 | 0.015666666666666666 | 0.0 | 0.0023333333333333335 | 0.006333333333333333 | 0.0 | 0.02266666666666667 | 0.0006666666666666666 | 0.0026666666666666666 | 0.0 | 0.0023333333333333335 | 0.0 | 0.0003333333333333333 | 0.021666666666666667 | 0.02 | 0.0 | 0.004666666666666667 | 0.1273333333333332 |
| FOS | 0.464 | 0.034333333333333334 | 0.137 | 0.017666666666666667 | 0.08433333333333333 | 0.0 | 0.009 | 0.022333333333333334 | 0.025333333333333333 | 0.012333333333333333 | 0.0 | 0.005666666666666667 | 0.007666666666666666 | 0.0 | 0.008 | 0.0036666666666666666 | 0.005666666666666667 | 0.0 | 0.002 | 0.0 | 0.0 | 0.043333333333333335 | 0.012333333333333333 | 0.0 | 0.002 | 0.10333333333333317 |
| Post | 0.11066666666666666 | 0.06333333333333334 | 0.19066666666666668 | 0.028 | 0.118 | 0.0 | 0.02 | 0.09866666666666667 | 0.039 | 0.021666666666666667 | 0.0 | 0.0016666666666666668 | 0.010666666666666666 | 0.0 | 0.007333333333333333 | 0.0033333333333333335 | 0.007 | 0.0 | 0.0 | 0.0 | 0.0 | 0.012 | 0.008666666666666666 | 0.0 | 0.0006666666666666666 | 0.2586666666666664 |
| Post | 0.14533333333333334 | 0.07166666666666667 | 0.20666666666666667 | 0.049 | 0.11466666666666667 | 0.0 | 0.014 | 0.06866666666666667 | 0.03966666666666667 | 0.012333333333333333 | 0.0 | 0.0026666666666666666 | 0.013333333333333334 | 0.0 | 0.008333333333333333 | 0.005666666666666667 | 0.01 | 0.0 | 0.0013333333333333333 | 0.0 | 0.0 | 0.027666666666666666 | 0.004 | 0.0 | 0.0013333333333333333 | 0.20366666666666677 |
| | None | None | None | None | None | None | None | None | None | None | None | None | None | None | None | None | None | None | None | None | None | None | None | None | None | None |
| Pre | 0.0 | 0.04566666666666667 | 0.03133333333333333 | 0.06466666666666666 | 0.041666666666666664 | 0.09 | 0.467 | 0.054 | 0.021333333333333333 | 0.0023333333333333335 | 0.021333333333333333 | 0.021 | 0.01633333333333333 | 0.04066666666666666 | 0.0 | 0.0 | 0.003 | 0.004333333333333333 | 0.0003333333333333333 | 0.0033333333333333335 | 0.0 | 0.0 | 0.0 | 0.0 | 0.0 | 0.07166666666666688 |
| Pre | 0.0006666666666666666 | 0.09233333333333334 | 0.07733333333333334 | 0.26866666666666666 | 0.020666666666666667 | 0.10766666666666666 | 0.15933333333333333 | 0.014666666666666666 | 0.049 | 0.006 | 0.03933333333333333 | 0.04133333333333333 | 0.02 | 0.005333333333333333 | 0.0 | 0.0 | 0.002 | 0.0016666666666666668 | 0.0 | 0.0023333333333333335 | 0.0 | 0.0 | 0.0 | 0.0013333333333333333 | 0.0 | 0.09033333333333327 |
| FOS | 0.0006666666666666666 | 0.029333333333333333 | 0.008333333333333333 | 0.015666666666666666 | 0.009666666666666667 | 0.12066666666666667 | 0.489 | 0.0016666666666666668 | 0.013333333333333334 | 0.0013333333333333333 | 0.059666666666666666 | 0.048666666666666664 | 0.013333333333333334 | 0.146 | 0.0 | 0.0 | 0.001 | 0.0006666666666666666 | 0.0003333333333333333 | 0.003 | 0.0016666666666666668 | 0.0 | 0.0 | 0.0 | 0.0 | 0.03600000000000003 |
| FOS | 0.0023333333333333335 | 0.014666666666666666 | 0.0023333333333333335 | 0.009 | 0.022333333333333334 | 0.17366666666666666 | 0.48533333333333334 | 0.0023333333333333335 | 0.004333333333333333 | 0.0003333333333333333 | 0.018333333333333333 | 0.049 | 0.008666666666666666 | 0.15233333333333332 | 0.0 | 0.0 | 0.0003333333333333333 | 0.0 | 0.0 | 0.005333333333333333 | 0.002 | 0.0 | 0.0 | 0.0 | 0.0 | 0.04733333333333345 |
| Post | 0.0023333333333333335 | 0.077 | 0.072 | 0.085 | 0.037 | 0.18666666666666668 | 0.17366666666666666 | 0.028 | 0.03966666666666667 | 0.003 | 0.06966666666666667 | 0.08633333333333333 | 0.02266666666666667 | 0.0013333333333333333 | 0.0 | 0.0 | 0.002 | 0.005666666666666667 | 0.0006666666666666666 | 0.002 | 0.0006666666666666666 | 0.0 | 0.0 | 0.0 | 0.0 | 0.10466666666666657 |
| Post | 0.0 | 0.097 | 0.029 | 0.16 | 0.03966666666666667 | 0.289 | 0.009 | 0.0016666666666666668 | 0.03966666666666667 | 0.005 | 0.11166666666666666 | 0.11033333333333334 | 0.03233333333333333 | 0.0 | 0.0 | 0.0 | 0.0023333333333333335 | 0.0 | 0.0 | 0.0 | 0.0 | 0.0003333333333333333 | 0.0 | 0.0 | 0.0 | 0.07299999999999995 |
| | None | None | None | None | None | None | None | None | None | None | None | None | None | None | None | None | None | None | None | None | None | None | None | None | None | None |
| Pre | 0.04566666666666667 | 0.035666666666666666 | 0.17233333333333334 | 0.04633333333333333 | 0.17166666666666666 | 0.04033333333333333 | 0.0 | 0.19266666666666668 | 0.035333333333333335 | 0.010666666666666666 | 0.0 | 0.004333333333333333 | 0.017 | 0.0 | 0.0 | 0.0016666666666666668 | 0.0013333333333333333 | 0.07733333333333334 | 0.0 | 0.0016666666666666668 | 0.0 | 0.0 | 0.0 | 0.0023333333333333335 | 0.0 | 0.1436666666666666 |
| Pre | 0.023666666666666666 | 0.11466666666666667 | 0.09666666666666666 | 0.112 | 0.167 | 0.09733333333333333 | 0.0 | 0.039 | 0.06333333333333334 | 0.03133333333333333 | 0.0 | 0.021 | 0.025 | 0.0 | 0.0 | 0.0 | 0.0033333333333333335 | 0.011 | 0.001 | 0.002 | 0.0006666666666666666 | 0.0 | 0.0 | 0.0 | 0.0 | 0.19099999999999995 |
| FOS | 0.5653333333333334 | 0.025 | 0.08566666666666667 | 0.011666666666666667 | 0.045 | 0.151 | 0.0 | 0.025 | 0.02033333333333333 | 0.010333333333333333 | 0.0 | 0.0026666666666666666 | 0.013666666666666667 | 0.0 | 0.0 | 0.0003333333333333333 | 0.004333333333333333 | 0.0003333333333333333 | 0.0 | 0.0 | 0.0 | 0.0 | 0.0 | 0.0 | 0.0 | 0.03933333333333322 |
| FOS | 0.4036666666666667 | 0.035333333333333335 | 0.13133333333333333 | 0.015 | 0.123 | 0.10266666666666667 | 0.0 | 0.029333333333333333 | 0.025 | 0.019666666666666666 | 0.0 | 0.0026666666666666666 | 0.014333333333333333 | 0.0 | 0.0 | 0.0003333333333333333 | 0.0023333333333333335 | 0.0006666666666666666 | 0.0006666666666666666 | 0.0 | 0.0 | 0.0 | 0.0 | 0.0 | 0.0 | 0.09399999999999997 |
| Post | 0.124 | 0.08933333333333333 | 0.15066666666666667 | 0.07633333333333334 | 0.15433333333333332 | 0.09366666666666666 | 0.0 | 0.009 | 0.058 | 0.03 | 0.0 | 0.004333333333333333 | 0.022333333333333334 | 0.0 | 0.0 | 0.001 | 0.001 | 0.0016666666666666668 | 0.001 | 0.0013333333333333333 | 0.0016666666666666668 | 0.0 | 0.0 | 0.0006666666666666666 | 0.0 | 0.17966666666666653 |
| Post | 0.059666666666666666 | 0.09133333333333334 | 0.15233333333333332 | 0.08866666666666667 | 0.207 | 0.07933333333333334 | 0.0 | 0.009333333333333334 | 0.05433333333333333 | 0.02266666666666667 | 0.0 | 0.002 | 0.024333333333333332 | 0.0 | 0.0 | 0.001 | 0.001 | 0.0036666666666666666 | 0.0003333333333333333 | 0.0006666666666666666 | 0.0 | 0.0 | 0.0 | 0.0003333333333333333 | 0.0 | 0.20200000000000007 |
| | None | None | None | None | None | None | None | None | None | None | None | None | None | None | None | None | None | None | None | None | None | None | None | None | None | None |
| Pre | 0.121 | 0.112 | 0.0013333333333333333 | 0.2713333333333333 | 0.07533333333333334 | 0.08233333333333333 | 0.0006666666666666666 | 0.01 | 0.041 | 0.019 | 0.0 | 0.02033333333333333 | 0.016 | 0.0 | 0.010666666666666666 | 0.0 | 0.009333333333333334 | 0.0016666666666666668 | 0.001 | 0.0 | 0.021 | 0.0 | 0.0 | 0.0 | 0.0 | 0.18599999999999983 |
| Pre | 0.221 | 0.11233333333333333 | 0.0026666666666666666 | 0.083 | 0.03933333333333333 | 0.071 | 0.030333333333333334 | 0.17366666666666666 | 0.015 | 0.014333333333333333 | 0.0 | 0.062 | 0.005333333333333333 | 0.0 | 0.07866666666666666 | 0.0006666666666666666 | 0.0023333333333333335 | 0.015666666666666666 | 0.0013333333333333333 | 0.0 | 0.0036666666666666666 | 0.0 | 0.0 | 0.0 | 0.0016666666666666668 | 0.06599999999999995 |
| FOS | 0.4846666666666667 | 0.044 | 0.013333333333333334 | 0.10166666666666667 | 0.028 | 0.08133333333333333 | 0.011333333333333334 | 0.008 | 0.014333333333333333 | 0.0033333333333333335 | 0.0 | 0.005333333333333333 | 0.006666666666666667 | 0.0 | 0.14666666666666667 | 0.0 | 0.008333333333333333 | 0.001 | 0.0 | 0.0013333333333333333 | 0.0016666666666666668 | 0.0 | 0.0 | 0.0 | 0.011666666666666667 | 0.02733333333333321 |
| FOS | 0.192 | 0.07533333333333334 | 0.037333333333333336 | 0.146 | 0.04733333333333333 | 0.098 | 0.093 | 0.08166666666666667 | 0.024333333333333332 | 0.012 | 0.0 | 0.014 | 0.010666666666666666 | 0.0 | 0.06966666666666667 | 0.0 | 0.007333333333333333 | 0.01 | 0.0023333333333333335 | 0.001 | 0.0003333333333333333 | 0.0 | 0.0 | 0.0 | 0.0026666666666666666 | 0.07500000000000007 |
| Post | 0.25166666666666665 | 0.12233333333333334 | 0.005 | 0.13533333333333333 | 0.029 | 0.082 | 0.0003333333333333333 | 0.0033333333333333335 | 0.011666666666666667 | 0.009666666666666667 | 0.0 | 0.023666666666666666 | 0.010333333333333333 | 0.0 | 0.0033333333333333335 | 0.0 | 0.006333333333333333 | 0.001 | 0.0003333333333333333 | 0.0 | 0.0003333333333333333 | 0.0 | 0.0 | 0.0 | 0.0023333333333333335 | 0.30200000000000027 |
| Post | 0.09433333333333334 | 0.11 | 0.035666666666666666 | 0.3413333333333333 | 0.06566666666666666 | 0.09466666666666666 | 0.0016666666666666668 | 0.035666666666666666 | 0.035333333333333335 | 0.021 | 0.0 | 0.006666666666666667 | 0.011333333333333334 | 0.0 | 0.0023333333333333335 | 0.0003333333333333333 | 0.010333333333333333 | 0.003 | 0.005 | 0.0006666666666666666 | 0.0003333333333333333 | 0.0 | 0.0 | 0.0 | 0.0016666666666666668 | 0.12300000000000011 |
### Chart
| Category | | | |
|---|---|---|---|d
c
Blautia
Bifidobacterium
*
*
*
*
*
**
Supplementary Figure S3. Changes in the relative abundance of bacteria in the fecal microbiota. a) The composition of microbiota in fecal samples using 16S rRNA sequencing. These figures were constructed by the top 25 bacteria at genus level respectively. b) PCoA analysis using weighted UniFrac distance of faecal microbiome colored by individuals (Left) and sampling periods (Right). c) The proportion of Bifidobacterium and Blautia in feces at genus level. * = padj <0.05, ** = padj <0.01, *** = padj <0.001. d) Shannon Index calculated from fecal microbiome.
Supplementary Figure S3

## Slide 7
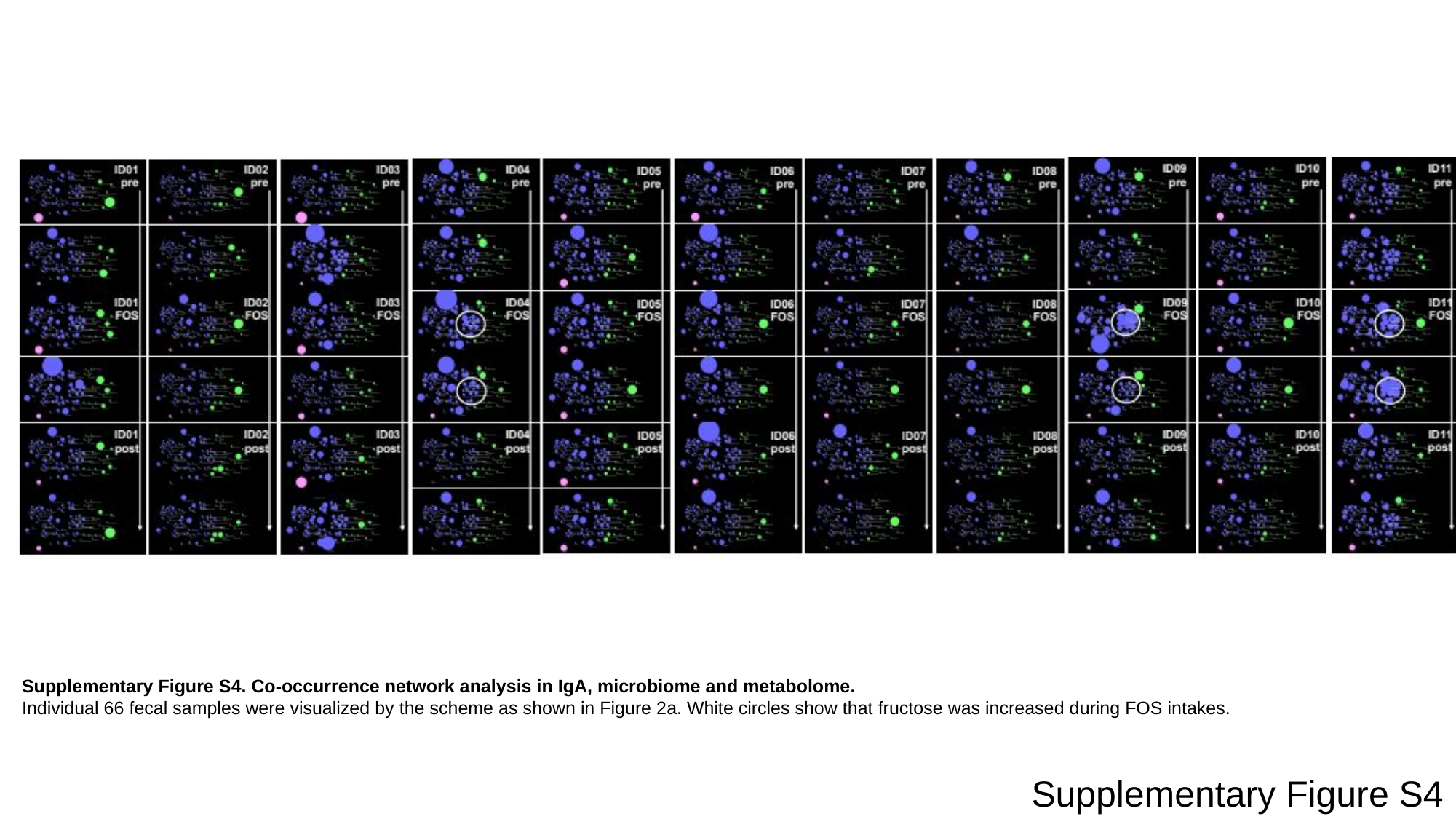

Supplementary Figure S4. Co-occurrence network analysis in IgA, microbiome and metabolome.
Individual 66 fecal samples were visualized by the scheme as shown in Figure 2a. White circles show that fructose was increased during FOS intakes.
Supplementary Figure S4
